# Defect‐Engineered CoSe_2_ Quantum Dots Exposing Highly Active (111) Facets with Lithiophilic‐Sulfurophilic Functionality for High‐Energy Lithium–Sulfur Batteries

**DOI:** 10.1002/advs.202511623

**Published:** 2025-11-20

**Authors:** Xue Li, Jiaqi Yu, Tianyu Jin, Yejing Li, Yaru Shi, Yong Jiang, Xiaoyu Liu, Shoushuang Huang, Kajsa Uvdal, Bing Zhao, Jiujun Zhang

**Affiliations:** ^1^ School of Environmental and Chemical Engineering Shanghai University/Shanghai Key Laboratory of Atomic Control and Application of Inorganic 2D Supermaterials Shanghai 200444 China; ^2^ College of Sciences/Institute for Sustainable Energy Shanghai University Shanghai 200444 China; ^3^ Division of Molecular Surface Physics and Nanoscience Department of Physics Chemistry and Biology Linköping University Linköping 58183 Sweden

**Keywords:** catalytic conversion, CoSe_2_ quantum dots, lithium polysulfides, lithium–sulfur batteries, redox kinetics

## Abstract

The shuttle of lithium polysulfides (LiPSs) and sluggish redox kinetics have posed significant barriers to advancing lithium–sulfur batteries. The design of lithiophilic‐sulfiphilic cathode show great promise, however, the integration of these multifunction through precise atomic‐level synergy remains a critical challenge. Herein, defective CoSe_2_ quantum dots (QDs) confined within carbon microspheres (CoSe_2_@C) are prepared as sulfur cathode host material. The exposure of the highly active (111) facets provides more active sites and accelerates the catalytic conversion kinetics of LiPSs. Additionally, the abundant selenium vacancies exhibit dual‐bonding capability with Li and S atoms, thereby improving the adsorption of LiPSs and lowering the reaction energy barrier. Moreover, the carbon microspheres matrix effectively alleviates the aggregation of CoSe_2_ QDs and increases the specific surface area. Benefiting from the above merits, the titled cathode exhibits enhanced conductivity and charge transfer, which effectively enhances the dynamics and alleviates shuttle effects. Consequently, the Li–S batteries assembled with CoSe_2_@C cathodes show extraordinary performance with an initial specific capacity of 1397 mAh g^−1^ at 0.2 C, a decay rate of 0.029% per cycle after 1000 cycles at 2 C. This work offers viewpoint for designing highly efficient catalysts for Li–S batteries.

## Graphical abstract

Defective CoSe_2_ QDs confined within carbon microspheres (CoSe_2_@C) are prepared as an advanced sulfur cathode host material. The exposure of highly active (111) facets and the abundant selenium vacancies not only provides more active sites but also accelerates the catalytic conversion dynamics and lowers the reaction energy barrier of Li–S batteries.

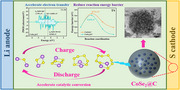

## 1 Introduction

With the continuous consumption of fossil energy, mankind is facing a serious energy and environmental crisis, and there is a higher demand for energy conversion and storage equipment.^[^ ^1^, ^2^ ^]^ Lithium–sulfur (Li–S) batteries, with their outstanding energy density (2600 Wh kg^−1^), excellent theoretical specific capacity (1675 mA h g^−1^), low cost, and environmentally friendly characteristics, have become an important development direction of the new generation of high‐energy‐density electrochemical energy storage and conversion systems.^[^ ^3^, ^4^ ^]^ However, the volume expansion of the sulfur cathode, the inherent insulation properties of the charge and discharge products, and the shuttle effect of LiPSs accelerate the degradation of battery capacity and cycle life, severely limiting its practical application and commercialization.^[^ ^5^ ^]^

To cope with these difficulties, researchers have devoted considerable efforts to developing efficient sulfur cathode host materials.^[^ ^6^ ^]^ Particularly, porous carbon materials with a large specific surface area and high electrical conductivity had been extensively explored as host materials for sulfur cathodes. Unfortunately, because of the weak physical adsorption of polysulfides by non‐polar carbon frameworks, the shuttle effect cannot be effectively suppressed.^[^ ^7^, ^8^, ^9^ ^]^ Recently, transition metal compounds such as oxides, nitrides, phosphides, sulfides, and selenides have attracted extensive attention due to their strong polarity and high catalytic activity.^[^ ^10^ ^]^ Among them, transition metal selenides (TMSes) are especially appealing, as their metal‐selenium bonds exhibit electron delocalization and higher electrical conductivity, providing a fundamental basis for accelerated catalytic kinetics, superior rate capability, and prolonged cycling stability. In recent years, various metal selenides have been explored as host materials of Li–S batteries. For example, Zhai et al.^[^ ^11^ ^]^ found that the MoSe_2‐x_@GA could promote the nucleation and dissociation of Li_2_S, regulate the uniform deposition of lithium, and inhibit the growth of lithium dendrites, leading to enhanced electrochemical properties. Zhou et al.^[^ ^12^ ^]^ synthesized a bimetallic selenide ZnSe/SnSe_2_, which selectively reduced the potential barrier during the reduction process. Shi et al.^[^ ^13^ ^]^ introduced N‐doping and Se‐vacancies into MoSe_2_ to promote bidirectional Li_2_S redox. Despite these merits, the weak contact between TMSes and substrates still limits charge transfer and diminishes catalytic activity.^[^ ^14^ ^]^ Therefore, integrating TMSes with conductive carbon substrates via a self‐assembly strategy enables fast electron transport and strong polysulfide anchoring, thus promoting LiPSs conversion and mitigating the shuttle effect.

Apart from improving conductivity and polysulfide adsorption, the insufficient density of active sites has emerged as another key bottleneck restricting the catalytic efficiency and overall performance of Li–S batteries.^[^ ^15^, ^16^ ^]^ Increasing the number and activity of active sites is therefore crucial to accelerate LiPSs conversion and enhance the performance of Li–S batteries. Recently, defect engineering has shown great promise as an effective strategy to modulate the electronic structure and conductivity of selenides, as well as to strengthen their chemical adsorption capability. For instance, Wang et al.^[^ ^17^ ^]^ introduced Se vacancies into MoSe_2_ catalyst to enhance the conductivity and reduce the dissociation energy barrier of Li_2_S. Wu et al.^[^ ^16^ ^]^ modulated the electronic structure of heterojunctions with Te vacancies to accelerate the redox conversion kinetics. Zhou et al.^[^ ^15^ ^]^ fabricated P defects by Mn doping to capture LiPSs. Xu et al.^[^ ^18^ ^]^ achieved the “adsorption‐directed migration‐catalytic” reaction mechanism for sulfur species by combining catalysts and sulfur vacancy adsorbents.

In addition to defect modulation, downsizing materials to the nanometer scale can further increase the density of active sites and enhance catalytic activity.^[^ ^19^ ^]^ Particularly, the high surface energy of quantum dots (QDs) can promote chemical anchoring of LiPSs, shorten the ion diffusion path, facilitates Li^+^ transport, lower the reaction barrier, and accelerates the catalytic conversion kinetics of LiPSs.^[^ ^20^ ^]^ Moreover, controlling the exposure of active crystal facets provides another efficient means to increase the number of accessible catalytic sites. For instance, Sun et al.^[^ ^21^ ^]^ demonstrated that sulfur‐passivated Mo_2_C (101) surfaces exhibited moderate chemical interactions with LiPSs, facilitating redox conversion during charge‐discharge processes. Jiang et al.^[^ ^22^ ^]^ exposed the {332} crystal plane of SnO_2_, which significantly reduced the decomposition energy barrier of Li_2_S. These advances suggest that designing cathodes with small size, high conductivity, abundant defects, and fully exposed active crystal planes, together with in‐depth exploration of their catalytic mechanisms, is essential for enhancing the catalytic conversion kinetics of LiPSs, suppressing the shuttle effect, and improving the overall performance of Li–S batteries.

Herein, CoSe_2_ QDs encapsulated by carbon microspheres (CoSe_2_@C) were designed as sulfur cathode host materials for Li–S batteries. The carbon microsphere encapsulation effectively alleviates the aggregation of CoSe_2_ QDs, improve the utilization rate of active sites, increase the conductivity of the sulfur cathode, shorten the electron/ion transport path, and improve the catalytic efficiency. The highly dispersed CoSe_2_ QDs ensure full exposure of active sites, thereby increasing the specific surface area and improving the interaction efficiency with LiPSs. Furthermore, the introduction of Se vacancies generates additional active sites that synergize with the QDs to form a dual‐function mechanism involving both chemical adsorption and catalytic conversion, thereby lowering the redox energy barrier and suppressing the shuttle effect of LiPSs. As a result, the Li–S battery assembled with CoSe_2_@C‐S cathode demonstrates excellent discharge capacity, rate performance, and durability. This work not only deepens the understanding of structure‐activity relationships in the design sulfur host materials but also opens new avenues for the development of high‐energy and long‐life Li–S batteries.

## 2 Results and Discussion

### 2.1 Synthesis and Characterization of CoSe_2_@C

**Figure 1:**
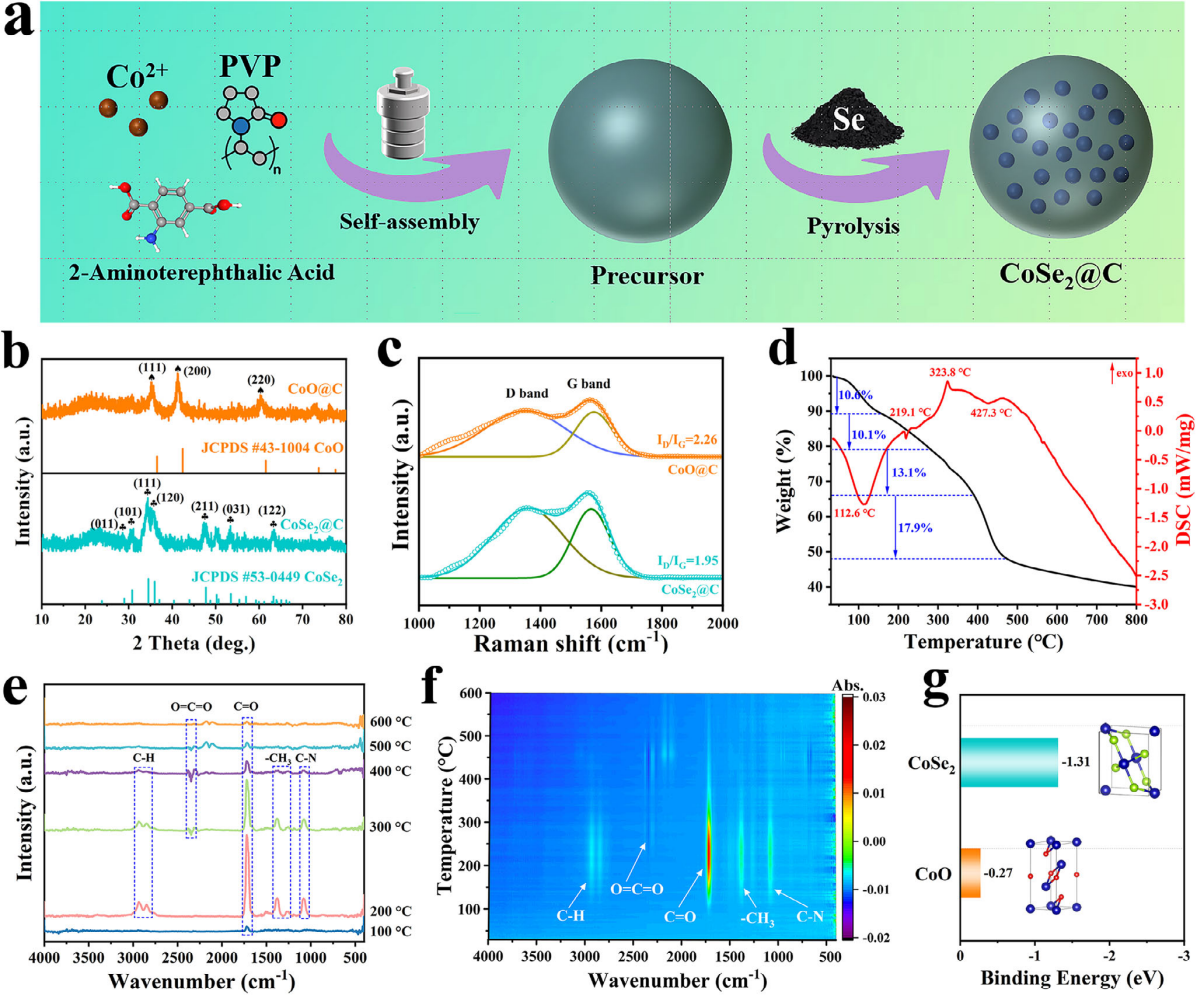
a) Schematic diagram of the synthesis of CoSe_2_@C catalyst. b) XRD patterns and c) Raman spectra of the as‐prepared CoSe_2_@C and CoO@C composites. d) TG‐DSC curves of the precursor and Se powder, e, f) TG‐FTIR spectra of the precursor powder. g) Binding energy of CoSe_2_ and CoO.

The CoSe_2_@C cathode host was synthesized by a typical solvothermal‐pyrolysis process, as illustrated in **Figure** **1**a. During the solvothermal process, 2‐aminoterephthalic acid acted as an organic ligand and reacted with Co^2+^ to form a MOFs (metal–organic framework) structure that was coated by PVP molecules. In the subsequent pyrolysis process, Se powder sublimated to produce selenium vapor, which combined with Co^2+^ to form CoSe_2_ QDs, accompanied by the decomposition of MOFs and the carbonization of PVP to form carbon microspheres, resulting in the formation of a well‐defined CoSe_2_@C composite. For comparison, the CoO@C was prepared by direct pyrolysis of precursor powder without Se powder. The XRD patterns of the obtained samples are exhibited in Figure 1b. The peaks appeared at ≈25° indicates the generation of carbon in the samples. Notably, the diffraction peaks of CoO@C can be well indexed to CoO (JCPDS #43‐1004). After the controlled selenization treatment, the diffraction peaks of the products correspond to the orthorhombic CoSe_2_ phase (JCPDS #53‐0449), confirming complete phase transformation from oxide to selenide. The enlarged XRD pattern of CoSe_2_@C is shown in Figure S1 (Supporting Information), and the intensity ratios of I(111)/I(101) and I(111)/I(120) are 2.53 and 1.35, respectively, compared with 2.23 and 1.13 in the standard PDF card. The enhanced intensity ratio indicates that the (111) crystal plane is the highly exposed crystal plane of CoSe_2_@C.

Generally, the organic ligands in MOFs will be carbonized to form conductive carbon layer during pyrolysis, which thereby contributes to enhancing the conductivity of electrode. To examine the structural characteristics of the resulting carbon, Raman spectroscopy was collected. As shown in Figure 1c, the I_D_/I_G_ ratio of CoSe_2_@C is 1.95, lower than that of CoO@C (2.26), suggesting that CoSe_2_@C has a higher degree of graphitization. It means that the selenization process can adjust the electronic structure of the carbon atoms and leads to a higher conductivity.^[^ ^23^ ^]^ The four‐point probe conductivity measurement results are shown in Figure S2 (Supporting Information). It can be clearly seen that the electronic conductivity of CoSe_2_@C is significantly higher than that of CoO@C. Besides, CoSe_2_@C presents a high Brunauer–Emmett–Teller (BET) surface area of 66.47 m^2^ g^−1^ with abundant mesoporous, which is conducive to exposing active site for electrocatalytic reaction (Figure S3, Supporting Information). Additionally, the pyrolysis process was investigated by thermogravimetric and differential scanning calorimetry (TG‐DSC) analysis(Figure 1d). From 30 to 155 °C, a mass loss of 10.6% corresponds to the volatilization of coordinated solvent molecules, while the 10.1% loss at 155–280 °C can be attributed to the sublimation of selenium powder. Additionally, the weight loss at 280–390 °C arises from the pyrolysis of MOFs precursor, and the final stage is associated with carbonization.^[^ ^24^ ^]^ The above TG–DSC results reveal that the MOF precursor decomposes in a selenium‐rich vapor environment, and allows the Co species released during pyrolysis to readily react with Se, ultimately leading to the formation of ultrasmall CoSe_2_ nanoparticles. Moreover, the rapid gas evolution during pyrolysis perturbs the crystallization process and disrupts the ordered arrangement of Co and Se atoms, leaving part of the selenium lattice sites unoccupied and generating Se vacancies.^[^ ^24^ ^]^ Thermogravimetric analysis‐Fourier Transform Infrared Spectrometry (TG‐FTIR) was further conducted to understand the pyrolysis process of the precursor (Figure 1e,f). The vibration peak of O═C═O bonds related to the pyrolysis of organic ligands first appears.^[^ ^25^ ^]^ The weak intensity of the O═C═O peak is due to the relatively small content of organic ligands. Therefore, during the precursor decomposition process, a part of the oxygen atoms in the organic ligands escape in the form of CO_2_, and the Co atoms and the remaining oxygen atoms combine to form CoO. Furthermore, the binding energy was calculated through density functional theory (DFT). The binding energy of CoSe_2_ (−1.31 eV) is remarkably lower than that of CoO (−0.27 eV) in Figure 1g, demonstrating that the binding of Co atoms to Se atoms is prior to O atoms, which verifies the above experimental results.

**Figure 2:**
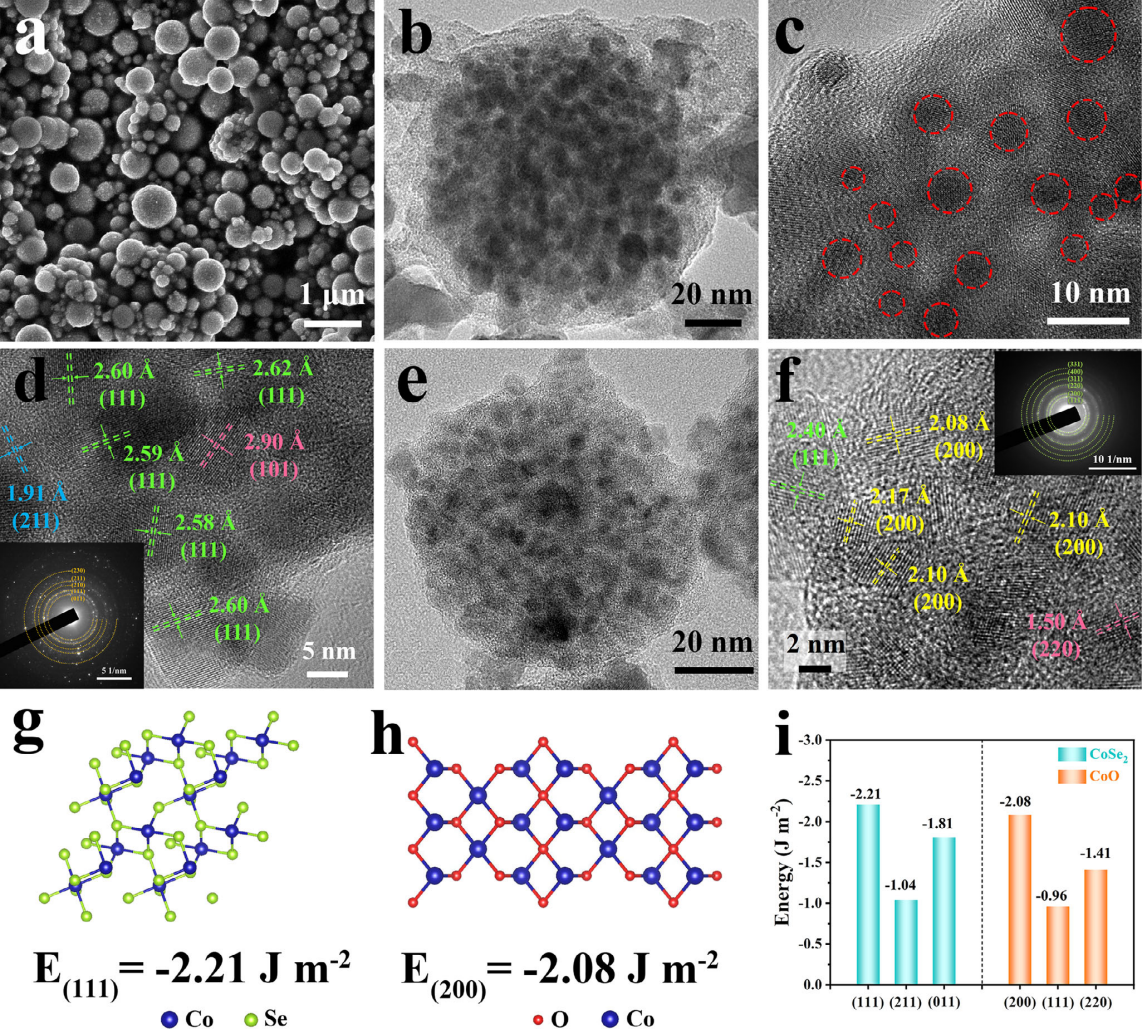
a) SEM, b,c) TEM images, d) HRTEM images of CoSe_2_@C. e) TEM images, f) HRTEM images of CoO@C. g–i) surface energy of CoSe_2_@C and CoO@C.

**Figure** **2**a, Figures S4, and S5 (Supporting Information) show the SEM and TEM images of CoSe_2_@C, where it is clearly observed that the particles have a spherical shape and the three elements of Co, Se, and C were evenly distributed. Subsequently, more detailed information on the structure of CoSe_2_@C was further investigated by HRTEM. In Figure 2b,c, it is definitely observed that numerous black dots of size 2–6 nm are encapsulated in amorphous carbon microspheres. The obvious lattice fringes of 2.58–2.62 Å correspond to the (111) crystal planes of CoSe_2_ (Figure 2d; Figure S6a,b, Supporting Information), which is the highly exposed crystal planes of CoSe_2_ QDs and consistent with the XRD result in Figure 1b. Furthermore, the surface energy of different crystal planes in CoSe_2_ is compared (Figure 2g–i; Figure S6c,d, Supporting Information), it can be seen that the (111) crystal plane has the lowest surface energy (−2.21 J m^−2^), indicating that this crystal plane has the highest catalytic activity. Similarly, Figure S7 (Supporting Information) and Figure 2e depicts the uniform distribution of Co, O, and C elements as well as the uniform distribution of CoO QDs in amorphous carbon microspheres. The (200) crystal plane is the highly exposed crystal surface of CoO QDs (Figure 2h), whose surface energy is −2.08 J m^−2^ (Figure 2i; Figure S8, Supporting Information), less than that of other crystal plane, indicating that this crystal plane is most stable.^[^ ^26^ ^]^ In addition, the SEM images of bare CoSe_2_, CoO, and PVP‐C are characterized as shown in Figure S9 (Supporting Information). The morphology of CoSe_2_ and CoO is spherical with smooth surface, and the PVP derived carbon is massive in morphology, which is seriously inconsistent with the morphology of quantum dots encapsulated in carbon microspheres of CoSe_2_@C and CoO@C. The above results manifest that the in situ formation of CoSe_2_ and CoO in the carbon matrix restricts the growth of nanoparticles in the process of PVP pyrolysis, leading to ultrafine QDs embedded in the carbon microspheres.^[^ ^26^ ^]^ Therefore, CoSe_2_ QDs encapsulated in carbon microspheres can significantly alleviate the aggregation of CoSe_2_ nanoparticles. The highly dispersed CoSe_2_ QDs can ensure the complete exposure of the active site and the active (111) crystal plane, effectively prevent the catalytic inactivation caused by the aggregation of the active site and significantly increases the specific surface area, which thereby enhances the interaction efficiency with LiPSs.

### 2.2 DFT Calculation and Chemisorption

**Figure 3:**
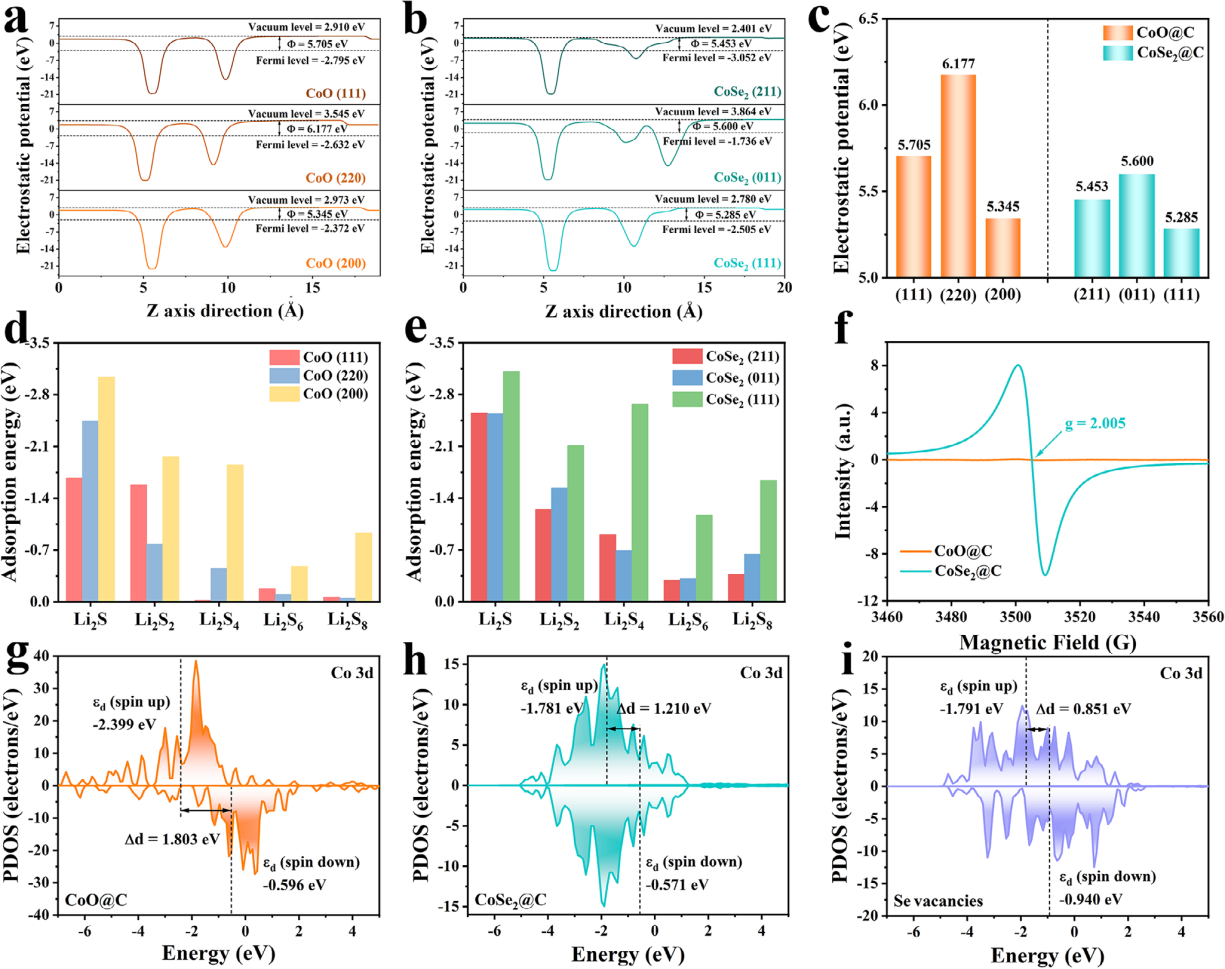
Calculated work function of various crystal planes of a) CoO@C and b) CoSe_2_@C. c) The value of the work function. The adsorption energy of various crystal planes of d) CoO@C and e) CoSe_2_@C. f) EPR spectra of CoO@C and CoSe_2_@C. PDOS for Co 3d orbitals of g) CoO@C, h) CoSe_2_@C, and i) CoSe_2_@C with Se vacancies.

To further verify the superiority of regulated crystal planes of different QDs, DFT calculations were performed. First, according to the characterization results, the structures of different crystal planes of CoO and CoSe_2_ matched with the C‐layer were modeled and optimized. **Figure** **3**a–c displays the calculated work functions of the various crystal planes of CoO@C and CoSe_2_@C. It is obvious that the work function of the (111) crystal face (5.285 eV) of CoSe_2_@C is not only smaller than that of the other crystal faces of CoSe_2_@C, but also smaller than the optimal (200) crystal face (5.345 eV) of CoO@C. This indicates that for the (111) crystal plane of CoSe_2_@C, the energy required for the electron to transition from the Fermi level to the vacuum level is the least, suggesting that the electrons escape most easily and thus more electrons are involved in the catalytic conversion of LiPSs. The adsorption energy of different crystal planes of CoO@C and CoSe_2_@C for LiPSs were calculated in Figure 3d,e. The highly exposed (200) crystal face of CoO@C has more negative adsorption energy on LiPSs than other crystal faces, and the same is true of the (111) crystal face of CoSe_2_@C, indicating that the highly exposed crystal face of QDs has stronger adsorption capacity and better anchoring effect on LiPSs.^[^ ^27^ ^]^ Therefore, the highly exposed (200) crystal plane of CoO is adopted to represent CoO QDs, and the highly exposed crystal plane of CoSe_2_ (111) is used to represent CoSe_2_ QDs for subsequent theoretical calculations. The electronic structure was characterized by electron paramagnetic resonance (EPR) spectroscopy (Figure 3f). No obvious signal can be observed in the EPR spectrum of CoO@C, indicating that the concentration of oxygen vacancies is negligible. However, for CoSe_2_@C, there is a strong signal at g = 2.005, which is caused by the capture of unpaired electrons by selenium vacancies. Interestingly, the content of Se vacancies can be adjusted by varying the pyrolysis temperature of the precursor MOFs, as shown in Figure S10 (Supporting Information). Obviously, the vacancy content gradually decreases with the increase of annealing temperature. The CoSe_2_@C pyrolyzed at 400 °C has the highest content of Se vacancies. A greater abundance of Se vacancies not only improves electrical conductivity but also introduces more catalytically active sites, thus promoting faster LiPSs conversion kinetics. ^[^ ^19^ ^]^ Figure 3g–i illustrate the PDOS for the Co 3d orbital centers in CoO@C, CoSe_2_@C, and CoSe_2_@C with Se vacancies. The difference between the two d‐band centers of Co spin up and spin down is the *d* band center gap (Δ*d*). The Δ*d* of CoSe_2_@C with Se vacancies is 0.851 eV, which is less than the Δ*d* of CoO@C (1.803 eV) and CoSe_2_@C (1.210 eV). A smaller Δ*d* is conducive to the electron transfer in redox reactions.^[^ ^28^ ^]^

**Figure 4:**
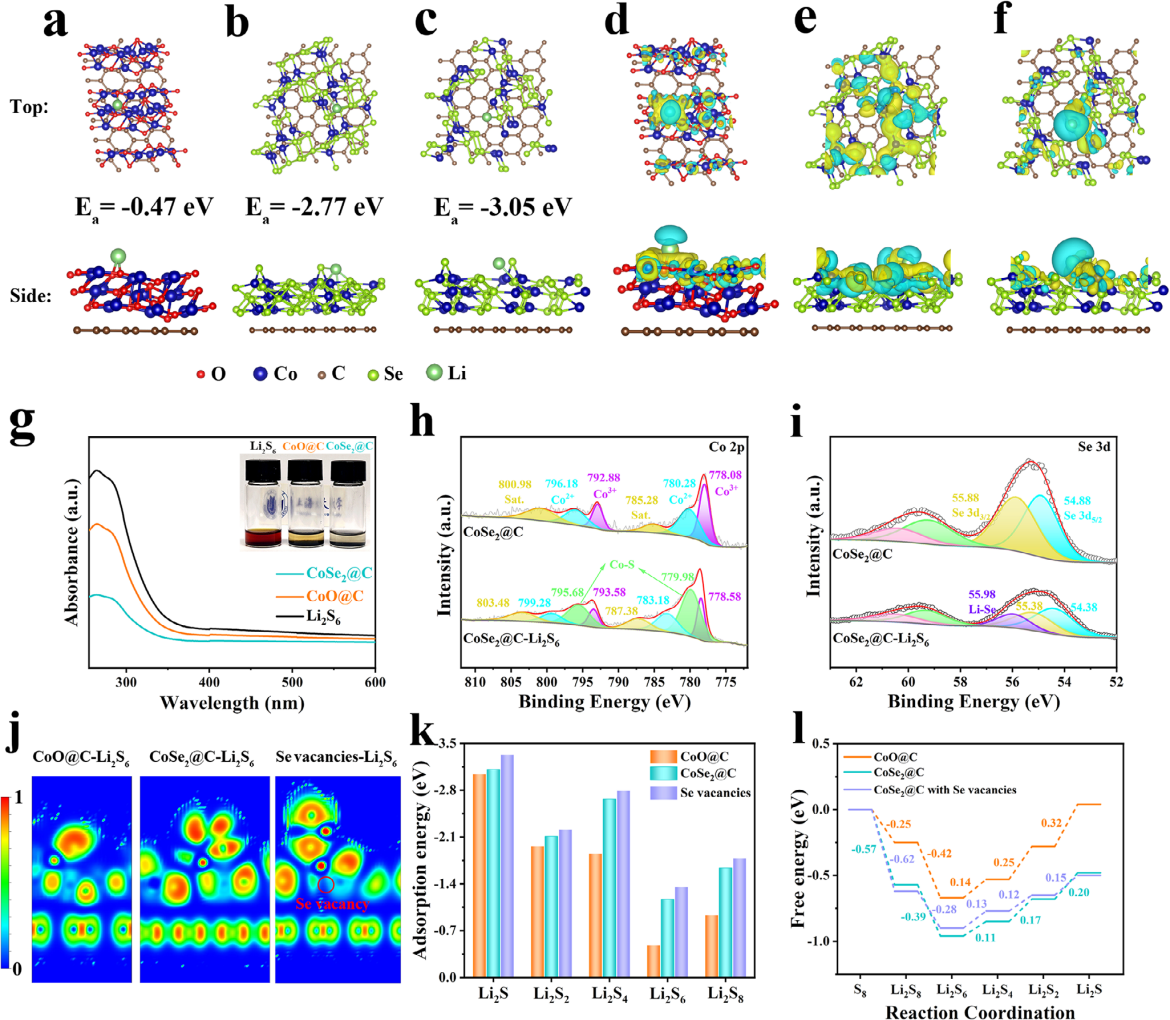
Optimized adsorption structures of Li atom absorbed on a) CoO@C, b) CoSe_2_@C, and c) CoSe_2_@C with Se vacancies. Calculated charge density differences of Li absorbed on d) CoO@C, e) CoSe_2_@C, and f) CoSe_2_@C with Se vacancies. g) UV–vis spectra and optical photograph of Li_2_S_6_ solution after standing for 6 h. High‐resolution XPS spectra of h) Co 2p and i) Se 3d of CoSe_2_@C before and after Li_2_S_6_ adsorption. j) Calculated electron locational function (ELF) of Li_2_S_6_ absorbed on CoO@C, CoSe_2_@C, and CoSe_2_@C with Se vacancies. k) Adsorption energy between LiPSs and CoO@C/CoSe_2_@C/CoSe_2_@C with Se vacancies. l) The Gibbs free energy profiles of LiPSs on CoO@C, CoSe_2_@C, and CoSe_2_@C with Se vacancies.

To get insight into the difference in the nucleophilic abilities of CoSe_2_@C and CoO@C, the adsorption energy of Li atom with different samples was calculated by DFT. As can be seen in **Figure** **4**a–c, the adsorption of CoSe_2_@C with Se vacancies (−3.05 eV) is much stronger than that of CoO@C (−0.47 eV) and CoSe_2_@C (−2.77 eV), demonstrating that the Se vacancies contribute to the stronger lithium affinity of CoSe_2_@C and conducive to enhancing the binding energy of LiPSs. The charge density difference was calculated to further clarify the charge transfer between Li atoms and two samples in Figure 4d–f, where yellow represents charge accumulation and blue represents charge depletion. It is noteworthy that there is a strong charge transfer between the Li and Se atoms, the charge density decreases around the Li atom and increases around the Se atom, while the change in charge density around the O atom is not significant. This conclusion can also be supported by the electron locational function (ELF) calculation in Figure S11 (Supporting Information), confirming the Li ions tend to adsorb on the surface of CoSe_2_ QDs, especially CoSe_2_ QDs with Se vacancies.^[^ ^29^ ^]^ The adsorption experiment was carried out by adding the same number of samples to Li_2_S_6_ solution to detect the adsorption capability of LiPSs. As shown in Figure 4g, the brown Li_2_S_6_ solution containing CoSe_2_@C became colorless after standing for 6 h. In contrast, the solution color is still yellow within CoO@C, indicating that CoSe_2_@C has a stronger ability to adsorb LiPSs. These results were confirmed by UV–vis spectroscopy, which clarified that the peak intensity of Li_2_S_6_ in the solution containing CoSe_2_@C was the lowest compared to the solution with the same number of CoO@C sample. The interaction between CoSe_2_@C/CoO@C and Li_2_S_6_ was then evaluated by XPS analysis. It can be seen from Figure 4h and Figure S12a (Supporting Information) that after the adsorption of Li_2_S_6_, the peaks of Co 2p of CoSe_2_@C and CoO@C both shifted toward higher binding energy, and Co─S bond appeared, which proves that Co is electrophilic and can anchor polysulfide ions.^[^ ^30^ ^]^ On the contrary, the Se 3d XPS spectrum of CoSe_2_@C‐Li_2_S_6_ in Figure 4i shifted to lower binding energy, and Li─Se bonds were generated, indicating an increase in electron density around Se atoms during LiPSs adsorption, which is in agreement with the calculation results in Figure 4e,f.^[^ ^16^ ^]^ However, the O 1s XPS spectrum of CoO@C‐Li_2_S_6_ shifted toward higher binding energy (Figure S12b, Supporting Information), which suggests that the nucleophilicity of O atoms is less than that of Se atoms. The 2D charge density of Li_2_S_6_ adsorbed on CoO@C, CoSe_2_@C, and CoSe_2_@C with Se vacancies is shown in Figure 4j. The CoSe_2_@C/CoSe_2_@C with Se vacancies adsorbed Li_2_S_6_ exhibited more distinct charge density compared to CoO@C, indicating a stronger interaction between CoSe_2_@C and Li_2_S_6_. Thanks to the presence of Se vacancies, there is a faster charge transfer between CoSe_2_@C and LiPSs, which effectively enhances the interface adsorption and catalytic activity.^[^ ^31^, ^32^ ^]^ Furthermore, the larger adsorption energy of CoSe_2_@C with Se vacancies toward each LiPSs than those of CoO@C and CoSe_2_@C depicts a stronger anchoring capacity for conversion intermediates (Figure 4k).^[^ ^32^ ^]^ To verify the catalytic activity of CoSe_2_@C and CoO@C for polysulfide conversion, the Gibbs free energy of LiPSs catalytic process was calculated. As shown in Figure 4l, the solid–solid conversion involving Li_2_S_2_ to Li_2_S is the decisive step of the whole reaction, and the *∆G* of CoSe_2_@C with Se vacancies at this step is 0.15 eV, which is smaller than 0.20 eV of CoSe_2_@C and 0.32 eV of CoO@C, demonstrating that CoSe_2_@C with Se vacancies features prominent catalytic activity. These DFT calculations indicate that Se vacancies are beneficial to improve the adsorption and catalytic transformation of LiPSs of CoSe_2_@C.^[^ ^33^ ^]^

### 2.3 Catalytic Conversion Kinetics of Redox Reactions of CoSe_2_@C

**Figure 5:**
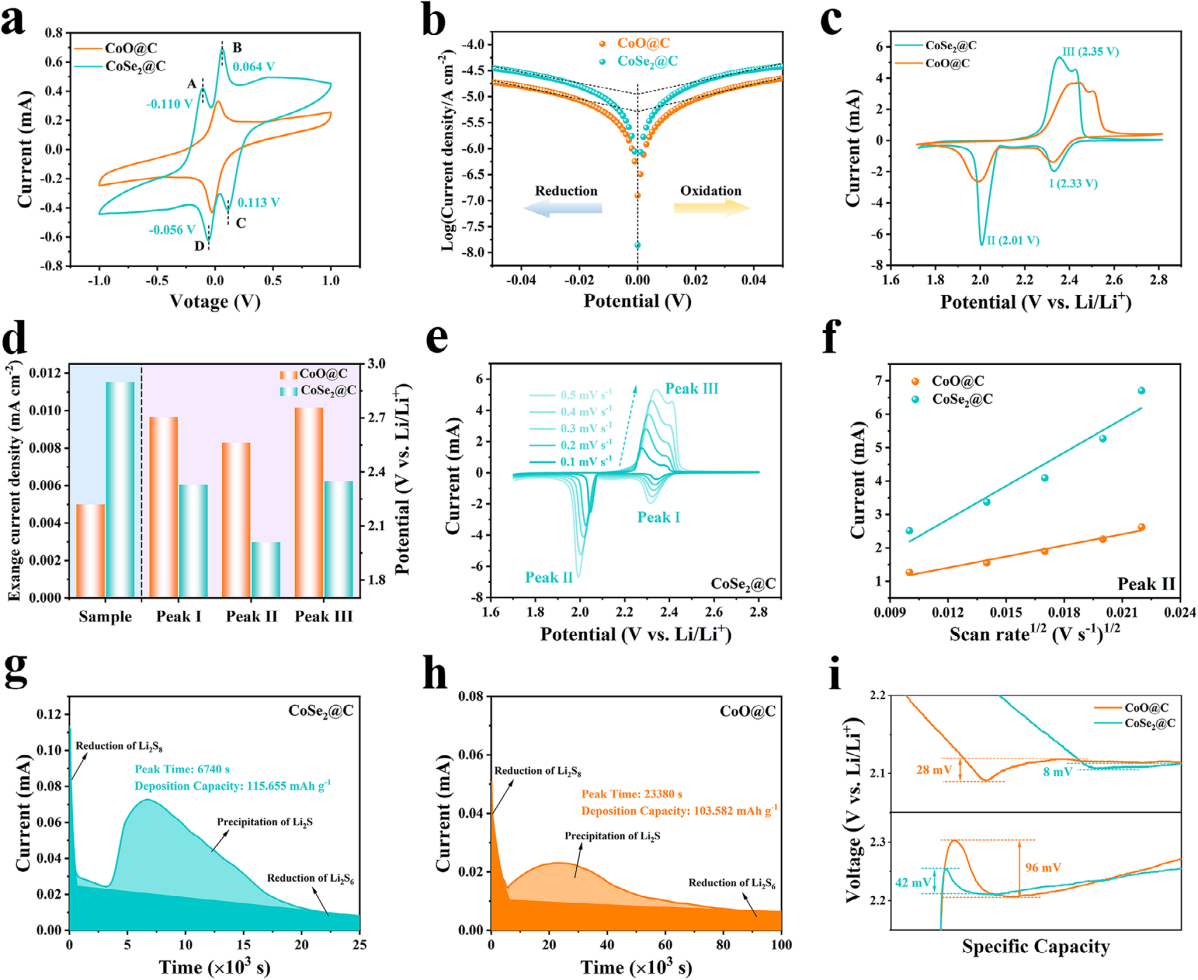
a) CV curves and b) Tafel slopes of symmetric cells. c) CV curves of Li–S batteries. d) The corresponding exchange current density and peak voltages from CV curves. e) CV plots with various scanning rates of CoSe_2_@C and f) peak current of the Peak II versus the square root of the scan rates. g,h) Nucleation tests of Li_2_S. i) Partially enlarged view of overpotentials for discharge and charge profiles.

The study of electrochemical kinetics is of great significance for evaluating catalytic performance. Symmetric batteries were assembled with Li_2_S_6_ electrolyte to study the catalytic ability of CoSe_2_@C on LiPSs. As shown in **Figure** **5**a, the cyclic voltammetry (CV) curves of CoSe_2_@C electrodes depict two pairs of distinct redox peaks at −0.110/0.113 V (peaks A/C) and 0.064/−0.056 V (peaks B/D). Peaks A and B are attributed to the reduction of Li_2_S_6_ to Li_2_S_4_ and Li_2_S_4_ to Li_2_S_2_/Li_2_S, respectively. Peaks C and D are related to the oxidation of Li_2_S_2_/Li_2_S to Li_2_S_4_ and Li_2_S_4_ to Li_2_S_6_, respectively.^[^ ^34^ ^]^ In contrast, the symmetrical cells with CoO@C electrodes exhibit only a pair of wider redox peaks and lower intensity, indicating that CoSe_2_@C has fast redox kinetics and excellent catalytic performance of LiPSs. In addition, the catalytic activity is further estimated by the exchange current density (Figure 5b), in which the CoSe_2_@C exhibits the lower Tafel slope and higher exchange current density of 0.012 mA cm^−2^ in comparison with CoO@C (0.005 mA cm^−2^), revealing that the CoSe_2_@C has rapid charge‐transfer rate and enhanced redox kinetics.^[^ ^35^ ^]^ TheCV curves of Li–S batteries based on two cathodes were tested within 1.7–2.8 V in Figure 5c. The CV curve of the battery with CoSe_2_@C cathode exhibited two obvious reduction peaks at 2.33 and 2.01 V (Figure 5c,d), representing the reduction of S_8_ to LiPSs and the subsequent transformation of LiPSs to solid Li_2_S_2_/Li_2_S, respectively. The two adjacent oxidation peaks at ≈2.35 V represent two successive oxidation processes, attributed to the conversion of Li_2_S_2_/Li_2_S to LiPSs and finally to S_8_.^[^ ^36^ ^]^ The potential difference between the two reduction peaks of CoSe_2_@C is significantly smaller than that of CoO@C, on behalf of the smaller polarization potential, suggesting that CoSe_2_@C significantly enhances the catalytic process of LiPSs. Moreover, CV curves at various rates were performed to clarify the Li^+^ diffusion behavior (Figure 5e; Figure S13, Supporting Information). Based on the Randles–Sevcik equation, the Li^+^ diffusion coefficient (*D*_Li_^+^) is proportional to the slope of the *I_p_*‐*v* ^1/2^ curves. As demonstrated in Figure 5f and Figure S14 (Supporting Information), the fitted *I_p_*‐*v* ^1/2^ slope and calculated the *D*_Li_^+^ of CoSe_2_@C is significantly higher than that of CoO@C, revealing that CoSe_2_@C has a greater Li^+^ diffusion rate, which is crucial for accelerating reaction dynamics and inhibiting shuttle effect of LiPSs.^[^ ^37^ ^]^

The Li_2_S deposition experiment was conducted to study the transformation kinetics from soluble LiPSs to solid Li_2_S. As shown in Figure 5g,h, CoSe_2_@C has a higher Li_2_S deposition capacity (115.655 mA h g^−1^) and an earlier response time (6740 s), in sharp contrast to CoO@C (103.582 mA h g^−1^ and 23 380 s), implying that CoSe_2_@C is favorable for the nucleation and precipitation of Li_2_S. Similarly, the higher capacity and earlier response time of CoSe_2_@C were also observed during Li_2_S dissolution process (Figure S15, Supporting Information). In addition, the overpotential of CoSe_2_@C during discharge and charge (Figure 5i, 24 mV, 42 mV) was much lower than that of CoO@C (28 mV, 96 mV), which is associated with the transformation of LiPSs to Li_2_S and Li_2_S to LiPSs, respectively. The decrease of polarization overpotential during Li_2_S conversion can further confirm the enhancement of LiPSs transformation kinetics of CoSe_2_@C.^[^ ^38^, ^39^ ^]^

**Figure 6:**
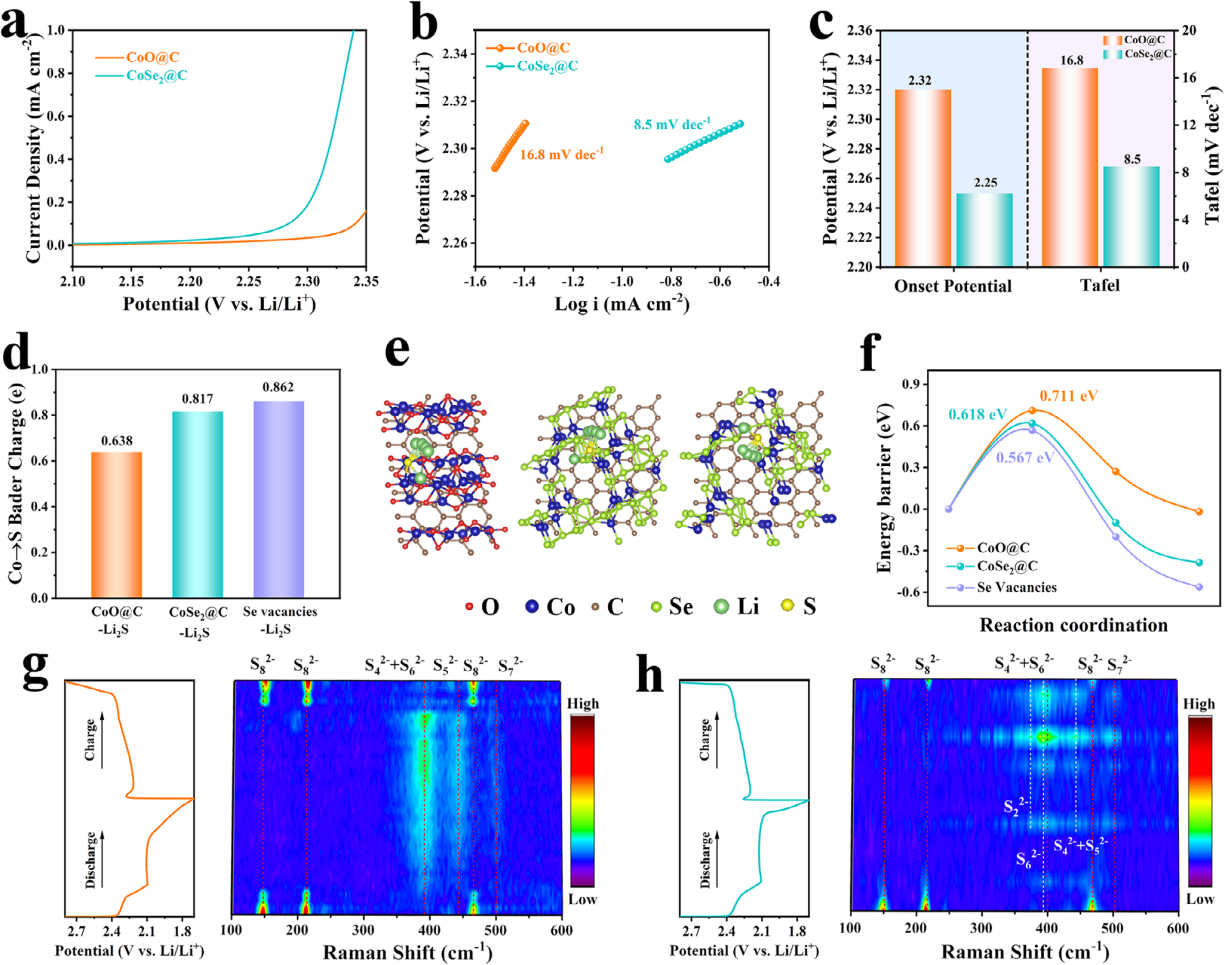
a) LSV tests and b) corresponding Tafel slopes of Li_2_S oxidation. c) Onset potentials and Tafel slopes from LSV tests. d) Bader charge of Co→S. e) The decomposed pathway of CoO@C, CoSe_2_@C, and CoSe_2_@C with Se vacancies, and f) the corresponding decomposition energy distribution of Li_2_S clusters. In situ Raman of g) CoO@C and h) CoSe_2_@C.

To further evaluate the kinetics of sulfur oxidation reaction, linear sweep voltammetry (LSV) measurements of Li–S batteries were carried out within the range of 1.7–2.8 V (**Figure** **6**a).^[^ ^40^ ^]^ The LSV curves depict that CoSe_2_@C is able to lower initial potential and higher current response than CoO@C, showing the faster Li_2_S decomposition reaction kinetics. This advantage is also confirmed by Tafel analysis (Figure 6b,c), where CoSe_2_@C provides a much lower Tafel slope of 8.5 mV dec^−1^ than that of CoO@C (16.8 mV dec^−1^). The above results exhibit that the well‐designed CoSe_2_@C can effectively promote the decomposition of Li_2_S, thereby enhancing the redox dynamics of Li–S batteries.^[^ ^41^ ^]^ Figure 6d shows the bader charge transfer between Li_2_S and CoO@C, CoSe_2_@C, and CoSe_2_@C with Se vacancies, and the charges transferred from Co to S are 0.638, 0.817, and 0.862, respectively. The result suggests that Se vacancies contribute to enhancing the interaction between CoSe_2_@C and Li_2_S. As the first step of charging process in Li–S battery, the decomposition barrier of Li_2_S on the catalyst surface is crucial for the analysis of the kinetics of Li_2_S oxidation and charging process.^[^ ^42^ ^]^ The decomposition pathways of Li_2_S on CoO@C and CoSe_2_@C and the corresponding decomposition barrier profiles are calculated in Figure 6e,f. The decomposition barrier of Li_2_S on the CoSe_2_@C with Se vacancies surface is 0.567 eV, which is smaller than that on the CoO@C surface (0.711 eV) and on the CoSe_2_@C surface (0.618 eV), which reveals that Li_2_S is more easily dissociated under the regulation of defective CoSe_2_ QDs catalytic center, thus accelerating the decomposition kinetics of Li_2_S.^[^ ^29^, ^34^ ^]^ Furthermore, in order to track the redox process and investigate the transformation mechanism of LiPSs, in situ Raman testing technique was performed. The in situ Raman spectra and corresponding charge and discharge profiles of the CoO@C and CoSe_2_@C electrodes are shown in Figure 6g,h. For the CoO@C electrode, the Raman peaks at 150, 213, and 468 cm^−1^ belong to S_8_^2−^, representing the reduction from solid S_8_ to long‐chain Li_2_S_8_. The S_8_^2−^ signals did not disappear until 2.1 V, accompanied by peaks of S_4_^2−^ and S_6_^2−^ at 390 cm^−1^, a peak of S_5_^2−^ at 443 cm^−1^, and a trace peak of S_7_^2−^ at 500 cm^−1^. These peaks of LiPSs persist throughout the discharge process as well as the charging process. When charging to 2.3 V, these peaks disappear and the peak of S_8_^2−^ begins to appear.^[^ ^43^ ^]^ These results suggest that the CoO@C electrode has a weaker catalytic effect on LiPSs and the reaction kinetics are slower. In sharp contrast, for the CoSe_2_@C electrode, when discharged to 2.1 V, the polysulfide signal appears at 400 cm^−1^, attributed to S_4_^2−^ and S_6_^2−^. As the discharge continues, the peaks appearing at 394, 446, and 504 cm^−1^ correspond to S_6_^2−^, S_4_^2−^+ S_5_^2−^, and S_7_^2−^, respectively, while the peaks appearing at 378 cm^−1^ belong to S_2_^2−^.^[^ ^44^ ^]^ In the process of charging, the peak of S_4_^2−^+S_6_^2−^, S_5_^2−^ appears when charging to 2.3 V, and the peak of S_7_^2−^ appears when charging to 2.35 V. Finally, a weak peak of S_8_^2−^ appears until 2.4 V, probably due to the oxidation of Li_2_S_8_ to S_8_. These results confirm that CoSe_2_@C can significantly reduce the existence time of LiPSs in Li–S batteries and effectively accelerate the reaction kinetics.^[^ ^27^ ^]^ The above experiments and theoretical results suggest that CoSe_2_@C can effectively strengthen the bidirectional electrocatalysis in sulfur oxidation and reduction to mitigate the shuttle effect and promote the utilization of sulfur.^[^ ^45^ ^]^

### 2.4 Electrical Performance of Li–S Batteries

**Figure 7:**
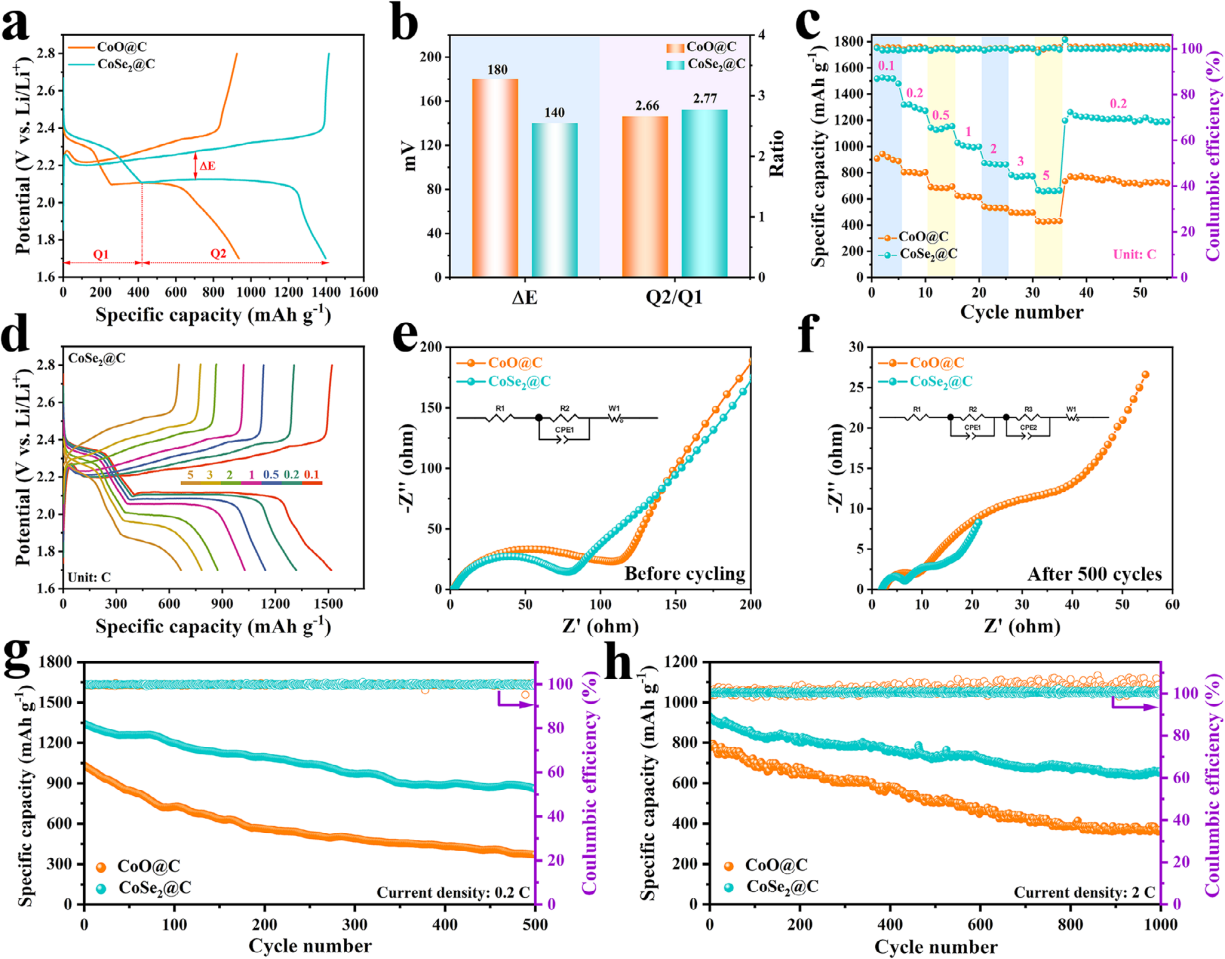
a) Charge–discharge curves of the Li–S batteries at 0.2 C. b) Values of ΔE and Q2/Q1. c) Rate capability. d) Charge–discharge curves at various rates of CoSe_2_@C. e,f) EIS spectra of before and after 500 cycles at 0.2 C. g,h) Cycling performance and the corresponding coulombic efficiency of the Li–S batteries with CoSe_2_@C and CoO@C cathodes at 0.2 C and 2 C.

The electrochemical properties were investigated to demonstrate the superiority of CoSe_2_@C in Li–S batteries. First, sulfur is infiltrated into the CoSe_2_@C nanostructure by a typical melting diffusion method. It can be seen from the thermogravimetric (TG) analysis in Figure S16 (Supporting Information) that the loading mass of sulfur is ≈75 wt.%. Next, the Li–S batteries were assembled with sulfur‐loaded cathodes, and the galvanostatic charge/discharge (GCD) curves of two different cathodes at 0.2 C are shown in **Figure** **7**a. The Li–S battery with CoSe_2_@C cathode has a higher specific capacity of 1397 mA h g^−1^ and a lower polarization potential of ΔE = 140 mV than CoO@C (936 mA h g^−1^, ΔE = 180 mV), implying the enhanced reaction kinetics. The GCD curves of samples with different Se vacancy contents show that Se vacancies contribute to achieving higher specific capacities, as shown in Figure S17 (Supporting Information). In addition, Q1 represents the ability of sulfur reduction for soluble LiPSs, Q2 is associated with LiPSs reduced to Li_2_S_2_/Li_2_S.^[^ ^31^, ^45^ ^]^ More importantly, the ratio of Q2/Q1 determines the electrocatalytic activity of LiPSs conversion reaction, and a higher Q2/Q1 represents better catalytic ability. In this case, the battery with CoSe_2_@C cathode showed a higher Q2/Q1 value (2.77) than CoO@C (2.66) (Figure 7b), confirming the outstanding catalytic conversion activity of CoSe_2_@C to LiPSs. To assess the advantages of CoSe_2_@C during the discharge–charge process, the rate capabilities and the corresponding charge/discharge profiles of different batteries were investigated at different rates every five cycles, as shown in Figure 7c,d and Figure S18 (Supporting Information). Figure 7c depicts that Li–S cell with CoSe_2_@C possesses the best rate characteristics, providing 1516, 1318, 1143, 1027, 873, 783, and 666 mA h g^−1^ discharge capacities at 0.1 C, 0.2 C, 0.5 C, 1 C, 2 C, 3 C and 5 C, respectively, when the current is switched back to 0.2 C, the discharge capacity can restore reversibly to 1361 mA h g^−1^, implying fast LiPSs conversion kinetics and excellent electrochemical structural stability.^[^ ^27^ ^]^ The electrochemical dynamics of two batteries with CoSe_2_@C and CoO@C cathodes was further studied by EIS (Figure 7e,f). In Figure 7e, the intercept of the semicircle intersecting the horizontal axis in the high frequency region represents the internal resistance (R_s_), the semicircle in the high frequency region belongs to the charge transfer resistance (R_ct_), and the slash line in the low frequency region relates to the diffusion impedance (Warburg impedance). However, the additional semicircle in the high frequency region can be seen in the batteries after the cycle in Figure 7f, which is associated with the interface resistance (R_surf_) of the insoluble Li_2_S_2_/Li_2_S passivation layer.^[^ ^36^, ^46^, ^47^ ^]^ The fitting results show that battery with CoSe_2_@C cathode exhibited smaller R_s_, R_ct_, and R_surf_ than CoO@C cathode both before and after cycling (Tables S1 and S2, Supporting Information). This clearly demonstrates the accelerated charge transfer dynamics of CoSe_2_@C batteries, which is beneficial to achieving large reversible capacity and cycle stability.^[^ ^41^ ^]^ The cycle performance of CoSe_2_@C batteries was evaluated at 0.2 C. As shown in Figure 7g, the Li–S battery with the CoSe_2_@C cathode displays a higher initial discharge capacity of 1343 mA h g^−1^ than the CoO@C cathode (1032 mA h g^−1^), and maintained a capacity of 865 mA h g^−1^ after 500 cycles, with only a minimal capacity degradation of 0.071% per cycle. The structure and composition of CoSe_2_@C largely remain after the cycling test (Figures S19–S21, Supporting Information), which suggests the superior electrochemical stability. To further demonstrate the superiority of CoSe_2_@C, the long cycle stability was tested at a high current density of 2 C (Figure 7h). After 1000 cycles, the battery with CoSe_2_@C cathode still has a high discharge capacity of 660 mA h g^−1^, depicting a high‐capacity retention rate (70.9%) and low‐capacity decay (0.029% per cycle). In sharp contrast, the battery with CoO@C cathode exhibit a more violent decay process and a poorer capacity retention rate (46.3%). Moreover, the specific capacity of 915 mAh g^−1^ can still be obtained with a high sulfur loading of 5 mg cm^−2^, and the capacity retention rate is 90% after 50 cycles (Figure S21, Supporting Information), indicating the rapid reaction kinetics and good electrochemical reversibility of CoSe_2_@C, which has the potential for application. It is worth noting that the outstanding cycling stability of the CoSe_2_@C cathode as well as the small decay rate are also obvious advantages over other reported sulfur cathode materials (Table S3, Supporting Information). Therefore, the CoSe_2_@C cathode can not only improve the utilization of active substances by improving the LiPSs transformation process, but also reduce the capacity decay and enhance the cycle stability by inhibiting the shuttle effect of LiPSs.^[^ ^33^, ^48^, ^49^ ^]^

## 3 Conclusion

In summary, CoSe_2_ QDs encapsulated by carbon microspheres were successfully constructed as sulfur cathode host material for Li–S batteries. The high graphitization degree of the carbon microspheres in CoSe_2_@C and the continuous electronic state at the Fermi level endow the CoSe_2_@C composite with high electronic conductivity. In addition, CoSe_2_ exists in the form of QDs in CoSe_2_@C, exposing more active (111) crystal planes, and increasing the specific surface area and surface energy of the catalyst, which is beneficial to the anchoring of LiPSs. The existence of selenium vacancies leads to strong charge transfer between CoSe_2_@C and LiPSs, which promotes the generation of Co─S and Li─Se bonds, increases the adsorption energy of LiPSs, and reduces the energy barrier of redox reaction. In situ Raman, series of electrochemical measurements and DFT calculations reveal that CoSe_2_@C can decrease the decomposition barrier of Li_2_S and promote the oxidation of Li_2_S, thereby improving the redox kinetics and alleviating shuttle effect of LiPSs. The Li–S batteries assembled with CoSe_2_@C‐S cathodes exhibit higher specific capacity (1397 mA h g^−1^) and lower polarization potential (ΔE = 140 mV) at 0.2 C, and an extremely long cycle life of 1000 cycles even at a high current density of 2 C, with only a minimal capacity degradation of 0.029% per cycle. This study not only clarifies the mechanism of enhancing the catalytic transformation of LiPSs of CoSe_2_@C, but also highlights its potential to improve the property of Li–S batteries. This work enriches the design prospects of catalytic host materials for sulfur cathodes.

## Conflict of Interest

The authors declare no conflict of interest.

## Supporting information



Supporting Information

## Data Availability

The data that support the findings of this study are available from the corresponding author upon reasonable request.
