# Supplementary material for: Defect‐Engineered CoSe2 Quantum Dots Exposing Highly Active (111) Facets with Lithiophilic‐Sulfurophilic Functionality for High‐Energy Lithium–Sulfur Batteries
Source: Adv Sci (Weinh). 2025 Nov 20;13(4):e11623. doi: 10.1002/advs.202511623 (PMC12822450; doi:10.1002/advs.202511623)
Supplement: Supplementary file 1 — Supporting Information [file ADVS-13-e11623-s001.docx]

Supporting Information

Defect-Engineered CoSe_2_ Quantum Dots Exposing Highly Active (111) Facets with Lithiophilic-Sulfurophilic Functionality for High-Energy Lithium-Sulfur Batteries

Xue Li, Jiaqi Yu, Tianyu Jin, Yejing Li, Yaru Shi, Yong Jiang,* Xiaoyu Liu, Shoushuang Huang,* Kajsa Uvdal,* Bing Zhao,* Jiujun Zhang

**Experimental Section**

**Synthesis of** **CoSe_2_@C and CoO@C Cathode Host**

CoSe_2_ quantum dots encapsulated by carbon microspheres (CoSe_2_@C) were prepared by a solvothermal and pyrolysis method. Briefly, Co(NO_3_)_2_**∙**6H_2_O (0.168 g), polyvinyl pyrrolidone (PVP) (0.25 g), and 2-aminoterephthalic acid (0.075g) were dispersed in 30 mL of N,N-Dimethylformamide (DMF), and heated to 150°C for 16 h. After cooling to room temperature, the suspension was centrifuged and washed with DMF several times and the black precursor powder was obtained after being dried. Subsequently, 50 mg of precursor powder and 150 mg of Se powder were placed in two ends of a porcelain boat, with the Se powder upstream of the tube furnace. The samples were heated to 400 °C for 1 h under Ar atmosphere at a heating rate of 2 °C min^-1^. After cooling to room temperature, the CoSe_2_@C was obtained. Similarly, CoO@C was prepared by heating without Se powder.

**Visual Adsorption Experiment of Li_2_S_6_**

To prepare the Li_2_S_6_ electrolyte, Li_2_S and S powders with a molar ratio of 1:5, were dissolved in a mixed solution of 1,3-dioxolane (DOL) and 1,2-dimethoxyethane (DME) (volume ratio 1:1) and stirred in a glove box at 60 °C for 12 h. The resulting Li_2_S_6_ electrolyte concentration was 0.2 M. The visual adsorption tests were carried out by immersing 20 mg of CoSe_2_@C and CoO@C powder into 5 mL pre-prepared Li_2_S_6_ solution as adsorbents, respectively.

**Symmetrical Cell Assembly and Measurements**

Symmetrical cells were assembled with two identical electrodes without sulfur loading as working electrode, 0.2 M Li_2_S_6_ solution as electrolyte, and Celgard 2400 PP as separator. cyclic voltammetry (CV) measurements were performed between -1.0 and 1.0 V at a scan rate of 5 mV s^-1^.

**Nucleation and Dissolution of Li_2_S**

Li_2_S and S powders with a molar ratio of 1:7 were added into tetraethylene glycol dimethyl etherand (TEGDME) stirred at 60 °C for 12 h to obtain 0.2 M Li_2_S_8_ electrolyte. 20 μL of Li_2_S_8_ electrolyte was dropped onto the cathode side, and 20 μL of electrolyte without Li_2_S_8_ was dropped onto the anode side. The assembled batteries were first discharged at 0.112 mA until the voltage dropped to 2.06 V, and then kept at 2.05 V constant potential discharge until the current dropped below 10^-5^ A to test Li_2_S deposition. Similarly, Li_2_S_8_ was completely converted to solid Li_2_S by constant discharge to 1.70 V at 0.1 mA and then to 1.80 V at 0.01 mA. The batteries were then charged at a constant potential at 2.40 V to oxidize the Li_2_S into soluble polysulfides.

**Fabrication of CoSe_2_@C-S and CoO@C-S Cathodes**

The sulfur cathodes were prepared via melt diffusion method. The host materials and sulfur powder were ground in a 3:7 weight ratio and heated at 155 °C for 12 h to obtain the sulfur composite. The obtained sulfur composite was mixed with Super P and polyvinylidene difluoride (PVDF) at a weight ratio of 8:1:1 in N-methyl-pyrrolidinone (NMP) to form a homogeneous slurry, followed by blading coating onto the aluminum current collector to prepare CoSe_2_@C-S and CoO@C-S cathodes with an active sulfur loading mass of 1.5 ~ 2 mg cm^-2^.

**Li-S Batteries Assembly and Electrochemical Performance Measurements**

Standard CR2032 coin batteries were assembled in an argon-filled glovebox via using lithium metal as anode, Celgard 2500 separators as separator, sulfur-containing electrodes as the cathode, and 1.0 M lithium bis(trifluoromethanesulfonyl)imide (LiTFSI) in a DOL/DME solution (1:1, v/v) with 2.0 wt% LiNO_3_ additive as the electrolyte, respectively. The galvanostatic discharge-charge tests were conducted at a voltage interval from 1.7 to 2.8 V using a NEWARE and LAND battery test system at 25 °C. The cells were aged for 10 h before testing and activated by 5 cycles at 0.1 C. The CV curves were collected in the potential range of 1.7 ~ 2.8 V at different scan rates ranging from 0.1 to 0.5 mV s^-1^. The electrochemical impedance spectroscopy (EIS) was recorded on electrochemistry workstation (Solartron analytical 1400 Cell Test System) at an open circuit potential with a scanning frequency of 100 kHz ~ 0.01 Hz.

**Materials Characterizations**

The crystal structure was characterized by X-ray diffraction (XRD) (Rigaku D/MAX2200) with a Cu-Kα radiation (λ =1.54059 Å). The morphology and elemental distribution were characterized by field emission scanning electron microscope (FESEM, JEOL, JSM-7500F) coupled with energy dispersive X-ray spectrometer and high-resolution transmission electron microscope (HRTEM, JEOL, JEM-2100F). The composition and valence states of the elements were identified by monochromatic Al Kα radiation X-ray photoelectron spectroscopy (XPS, Thermofisher, ESCALAB 250Xi). The visual adsorption experiment of LiPSs was measured by ultraviolet-visible spectrophotometer (PERSEE TU-1810). Thermogravimetric analysis (TGA, HTIACHISTA7300) was used to test sulfur loading. The in-situ Raman electrochemical cell (Hefei In-situ Technology. Co., Ltd.) was assembled from a quartz window, a perforated lithium foil, a perforated PP film, and S cathode. The Raman measurements were performed using a 532 nm laser with Raman spectrometer (Horiba LabRam HR Evolution).

**Theoretical Calculations**

The energy and electronic structure of the system were calculated using projected augmented waves (PAW) of density functional theory implemented in the Vienna Ab Initio Simulation Program Package (VASP). A plane-wave cutoff of 450 eV was used to completely relax the positions and lattice parameters of all atoms until the force acting on each atom was less than 0.03 eV Å^-1^. The Brillouin zone was sampled using the Monkhorst-Pack scheme, where a k-points mesh of 2 × 2 × 1 was used for structural relaxation in the Gamma-centered grids. The decomposition barrier of Li_2_S was calculated by climbing image-nudged elastic band (CI-NEB) to obtain the minimum energy path between a given initial and final position.


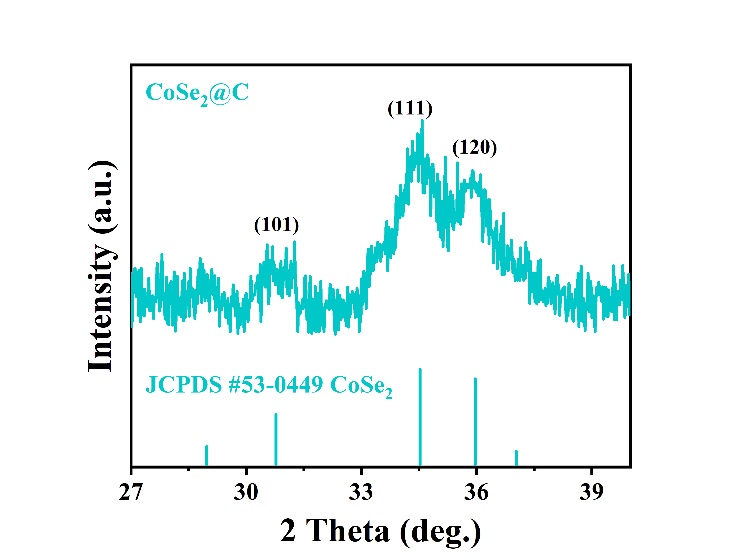


**Figure S1** Enlarged XRD pattern of CoSe_2_@C.


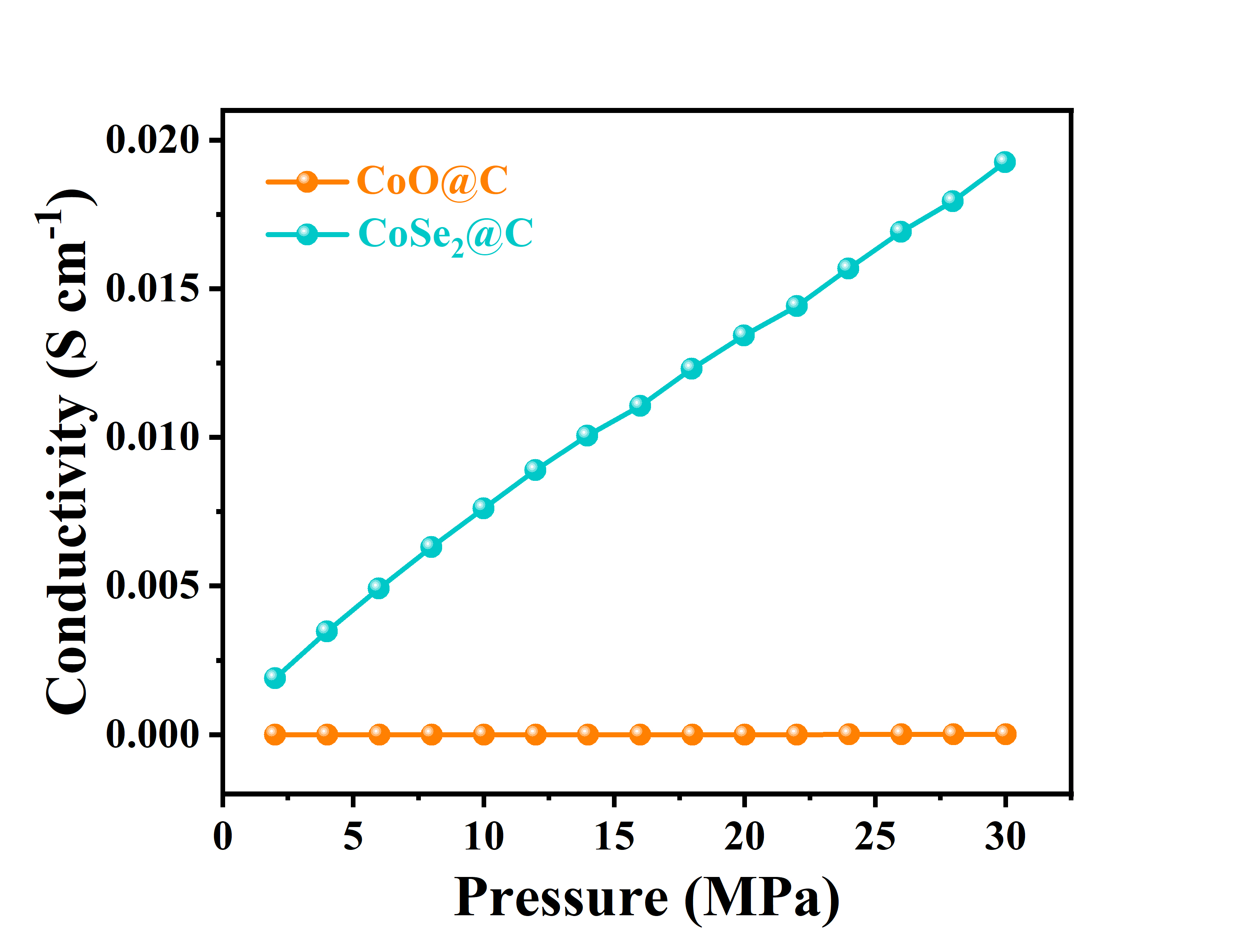


**Figure S2** The electronic conductivity of CoSe_2_@C and CoO@C at different pressure.


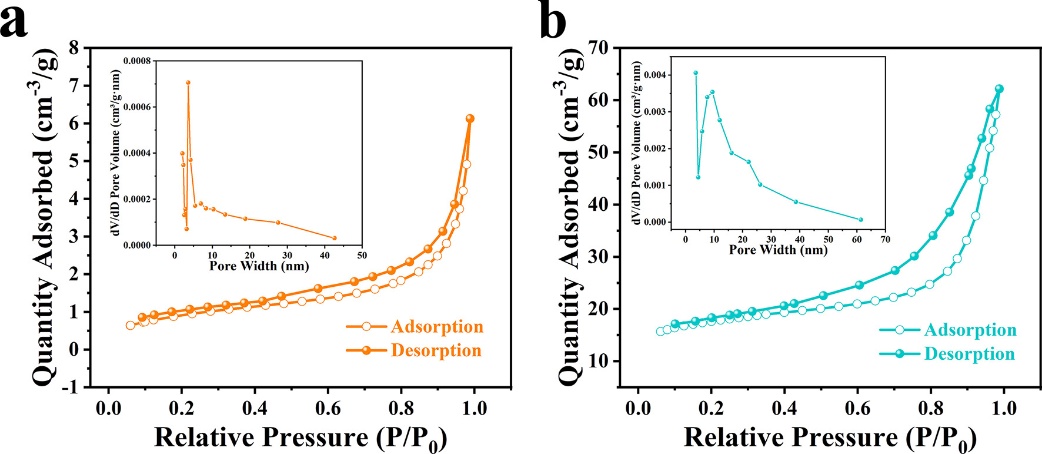


**Figure S3** N_2_ adsorption/desorption isotherm and pore size distribution (inset) of (a) CoO@C and (b) CoSe_2_@C.


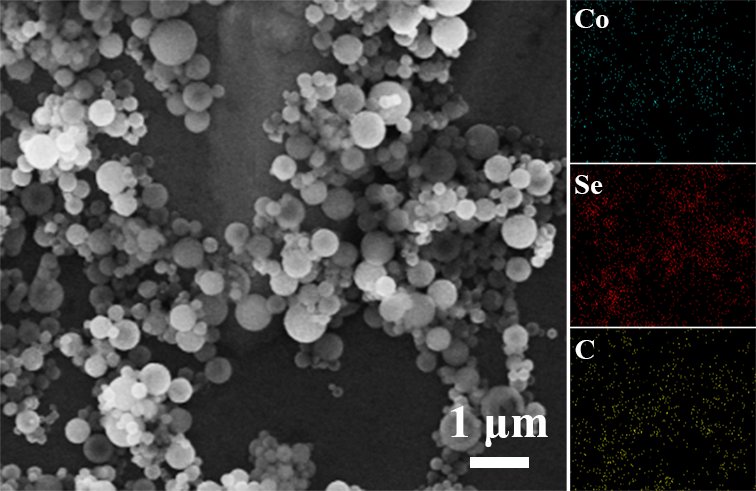


**Figure S4** SEM image and elemental mappings of CoSe_2_@C.


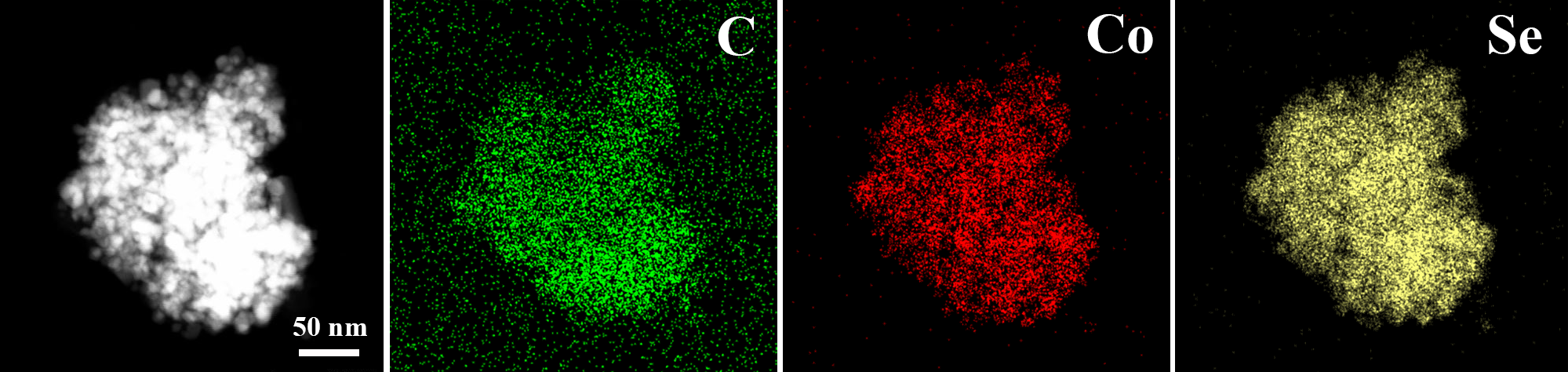


**Figure S5** TEM image and elemental mappings of CoSe_2_@C.


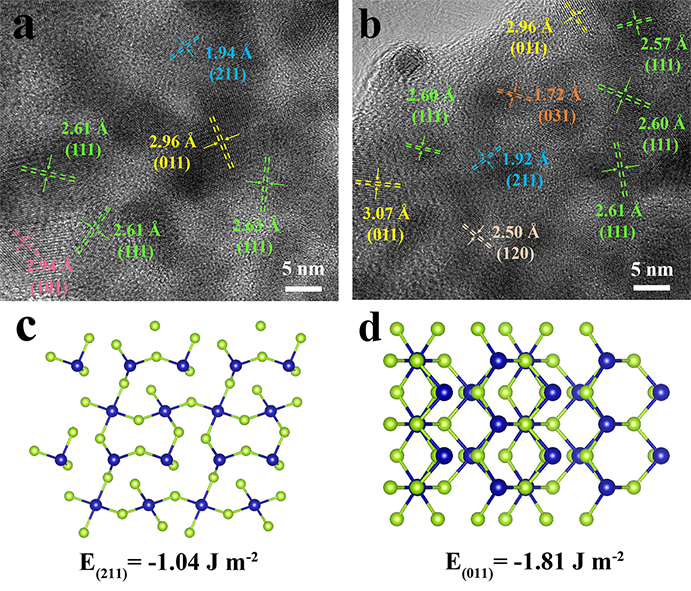


**Figure S6** HRTEM images and interface energy of CoSe_2_@C.


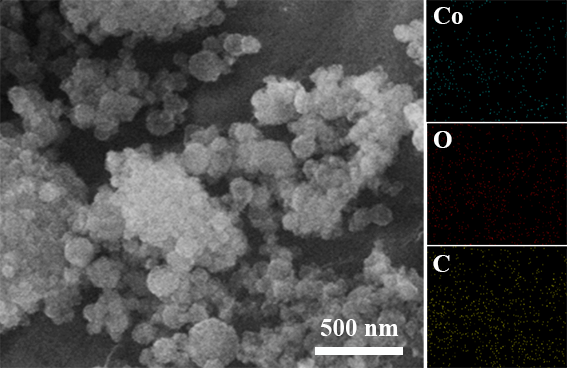


**Figure S7** SEM image and elemental mappings of CoO@C.


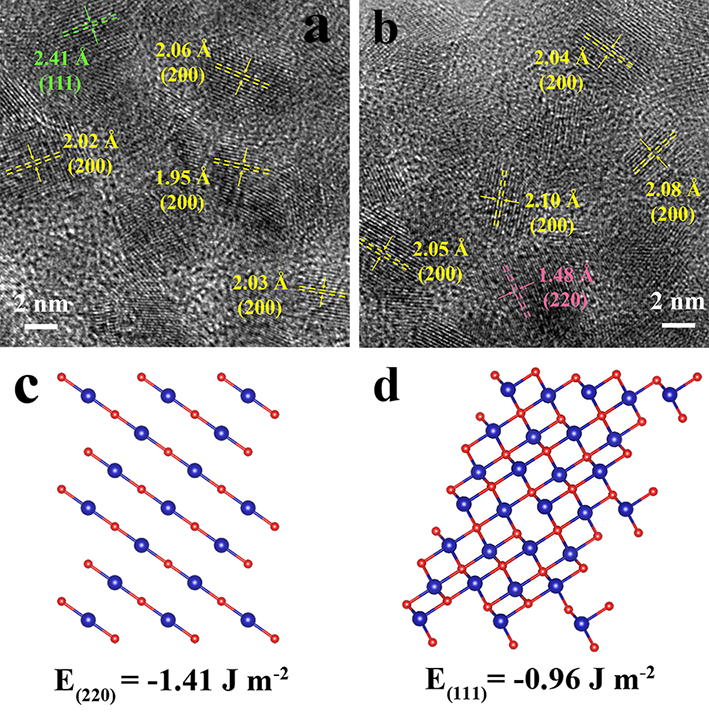


**Figure S8** HRTEM images and interface energy of CoO@C.


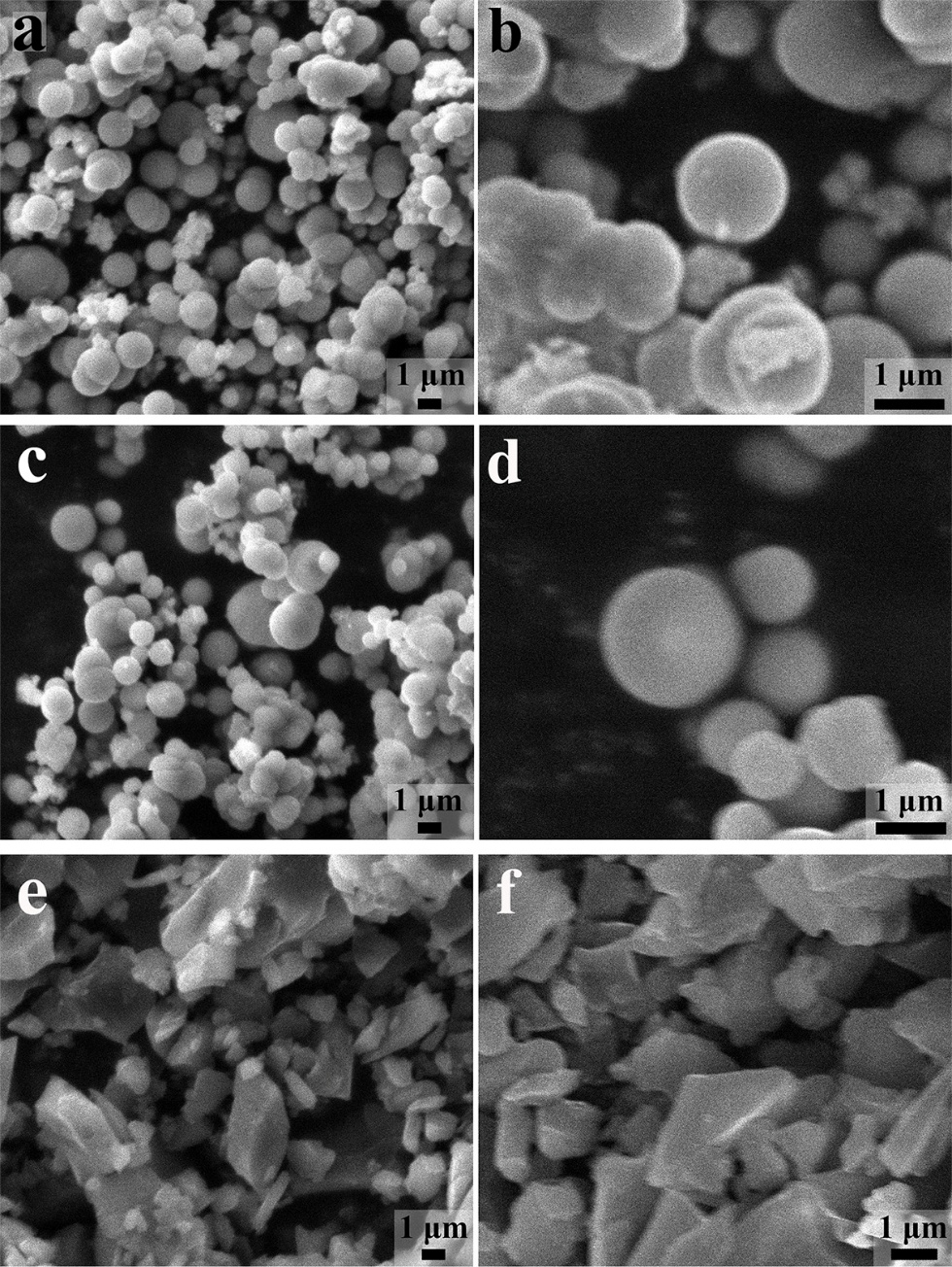


**Figure S9** SEM images of (a, b) CoSe_2_, (c, d) CoO, and (e, f) PVP-C.


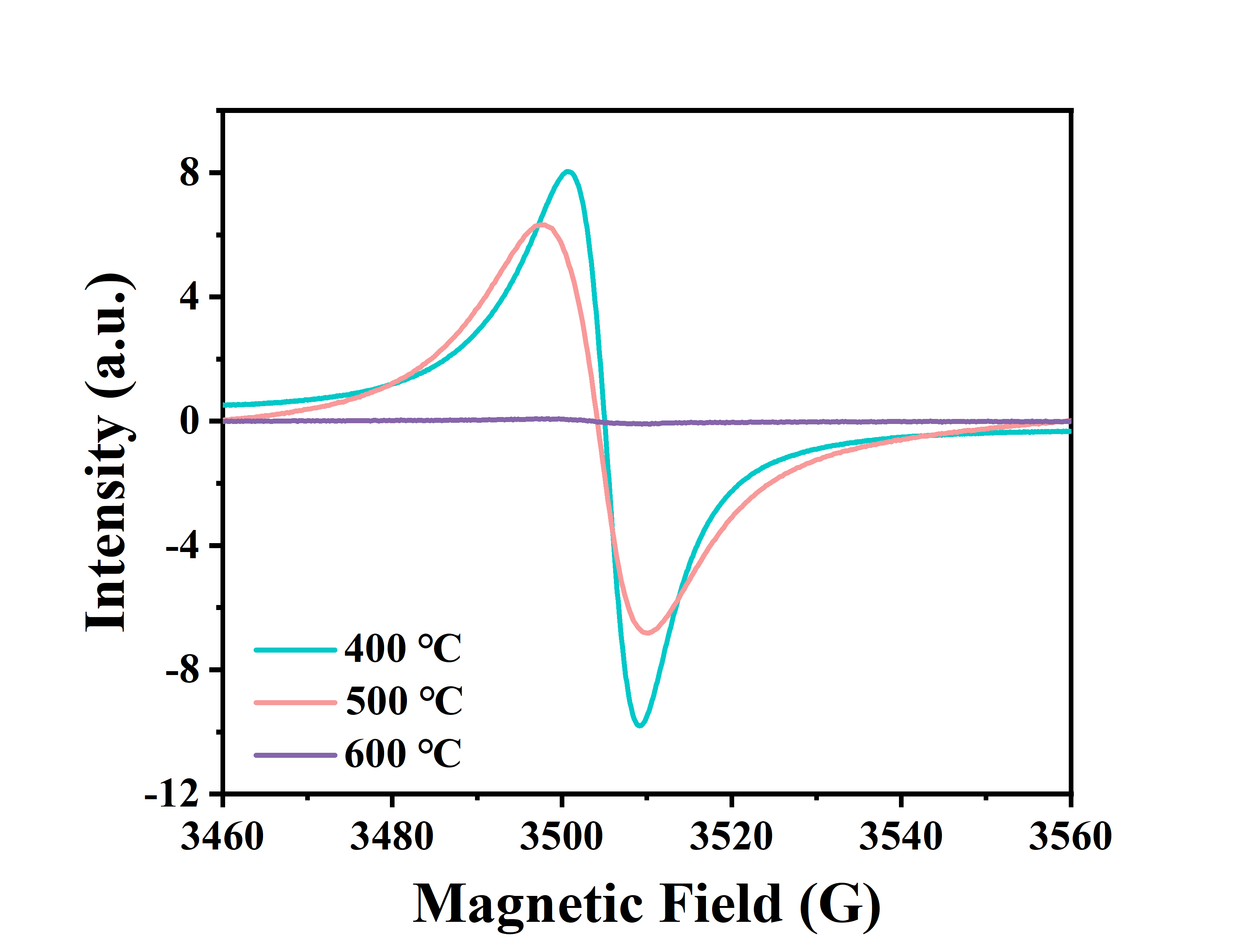


**Figure S10** EPR curves of the pyrolysis samples at different temperatures.


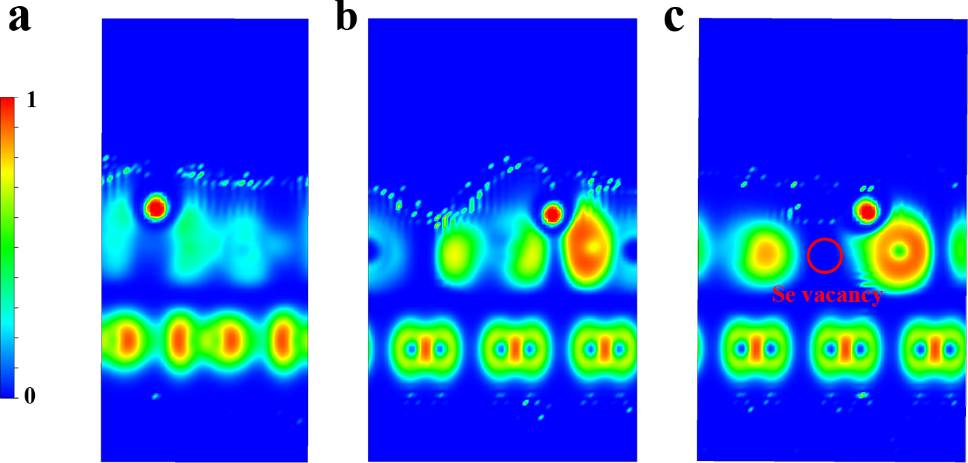


**Figure S11** Electron locational function of Li atom absorbed on (a) CoO@C, (b) CoSe_2_@C, and (c) CoSe_2_@C with Se vacancies.


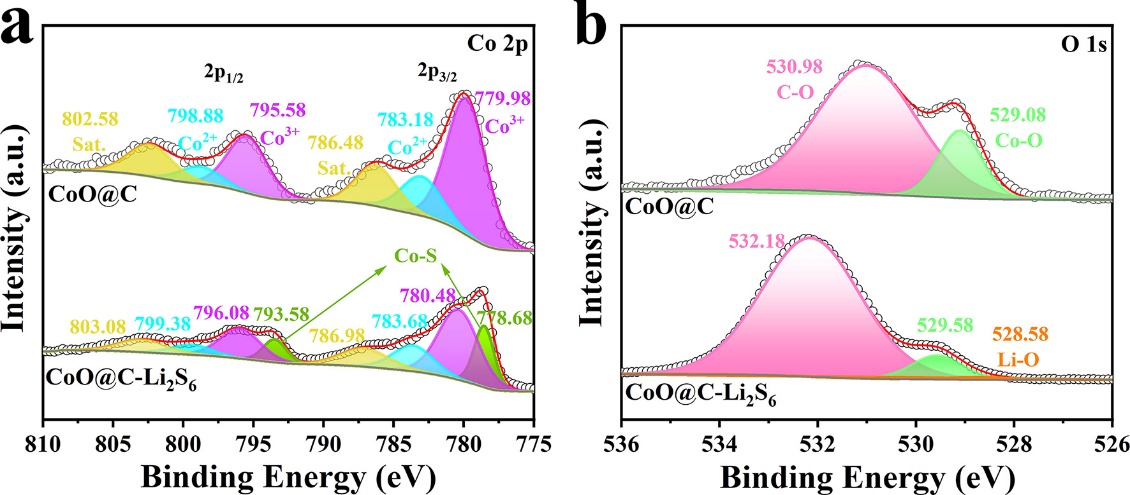


**Figure S12** High-resolution XPS spectra of **a** Co 2p and **b** O 1s of CoO@C before and after Li_2_S_6_ adsorption.


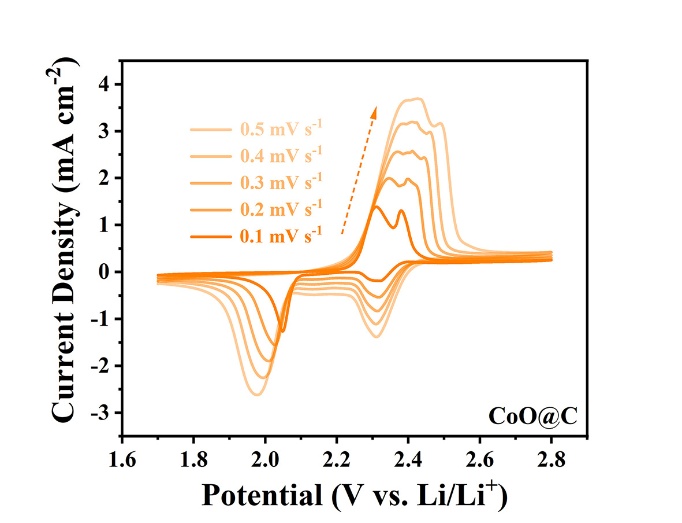


**Figure S13** CV curves at different scanning rates of CoO@C.


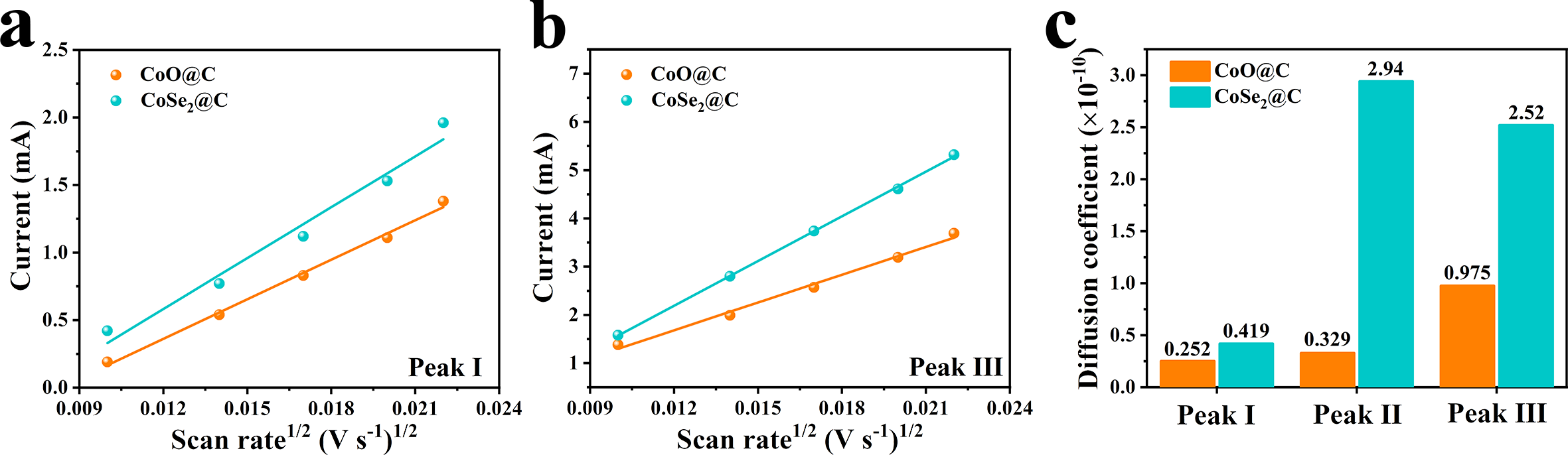


**Figure S14** Peak current of the (a) Peak Ⅰ and (b) Peak Ⅲ *vs*. the square root of the scan rates for CoSe_2_@C and CoO@C. (c) Li^+^ diffusion coefficient (*D*_Li_^+^).


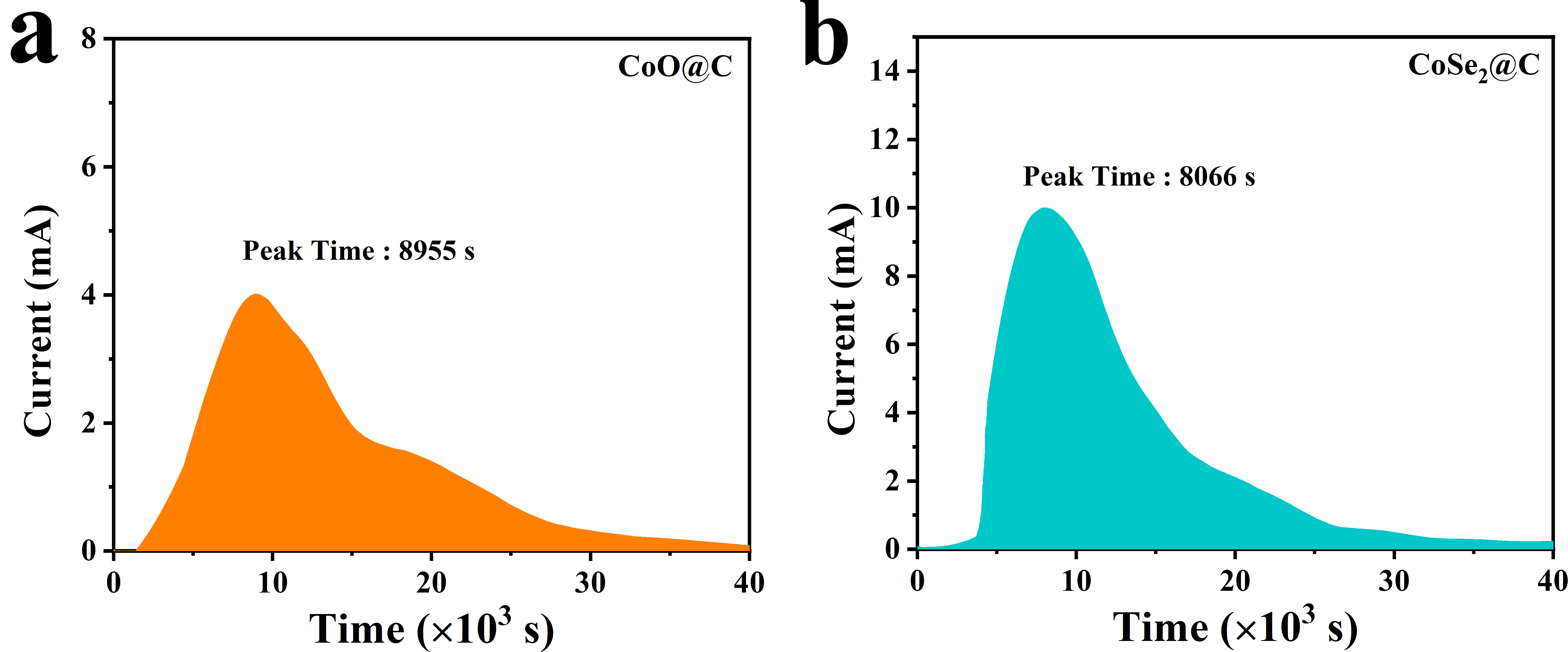


**Figure S15** Li_2_S decomposition of (a) CoO@C and (b) CoSe_2_@C.


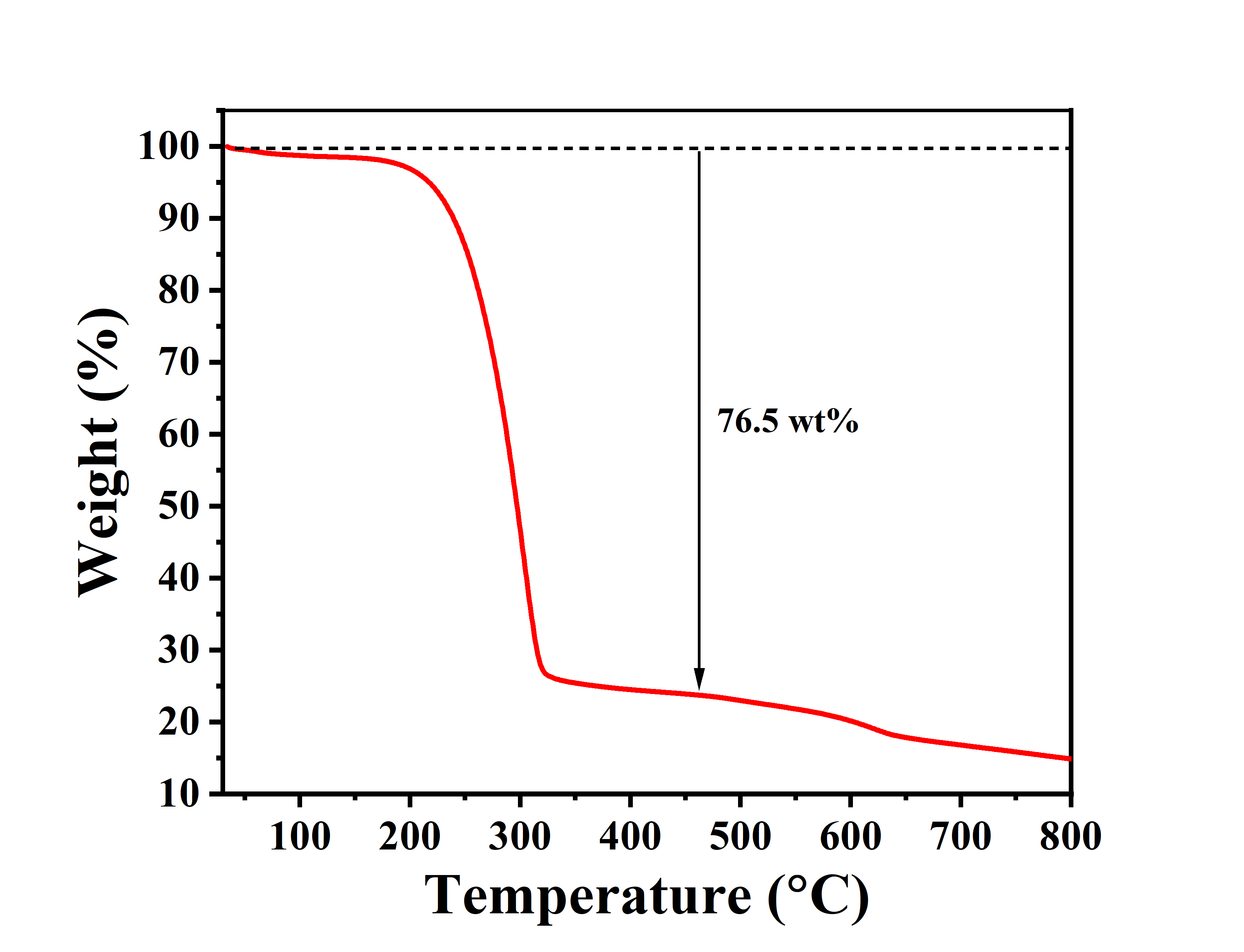


**Figure S16** TG curve of CoSe_2_@C-S.


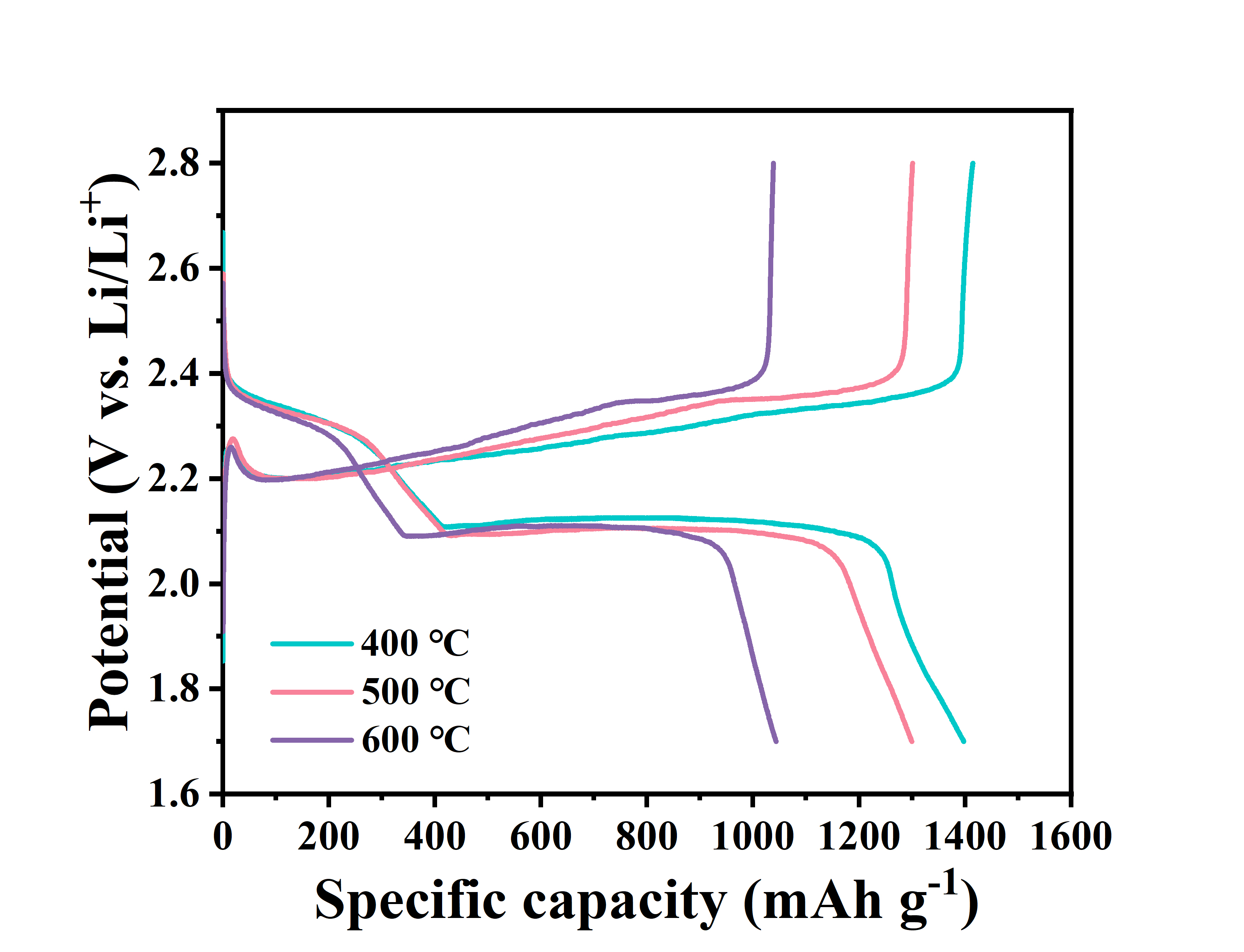


**Figure S17** GCD curves of the samples with different Se vacancy contents.


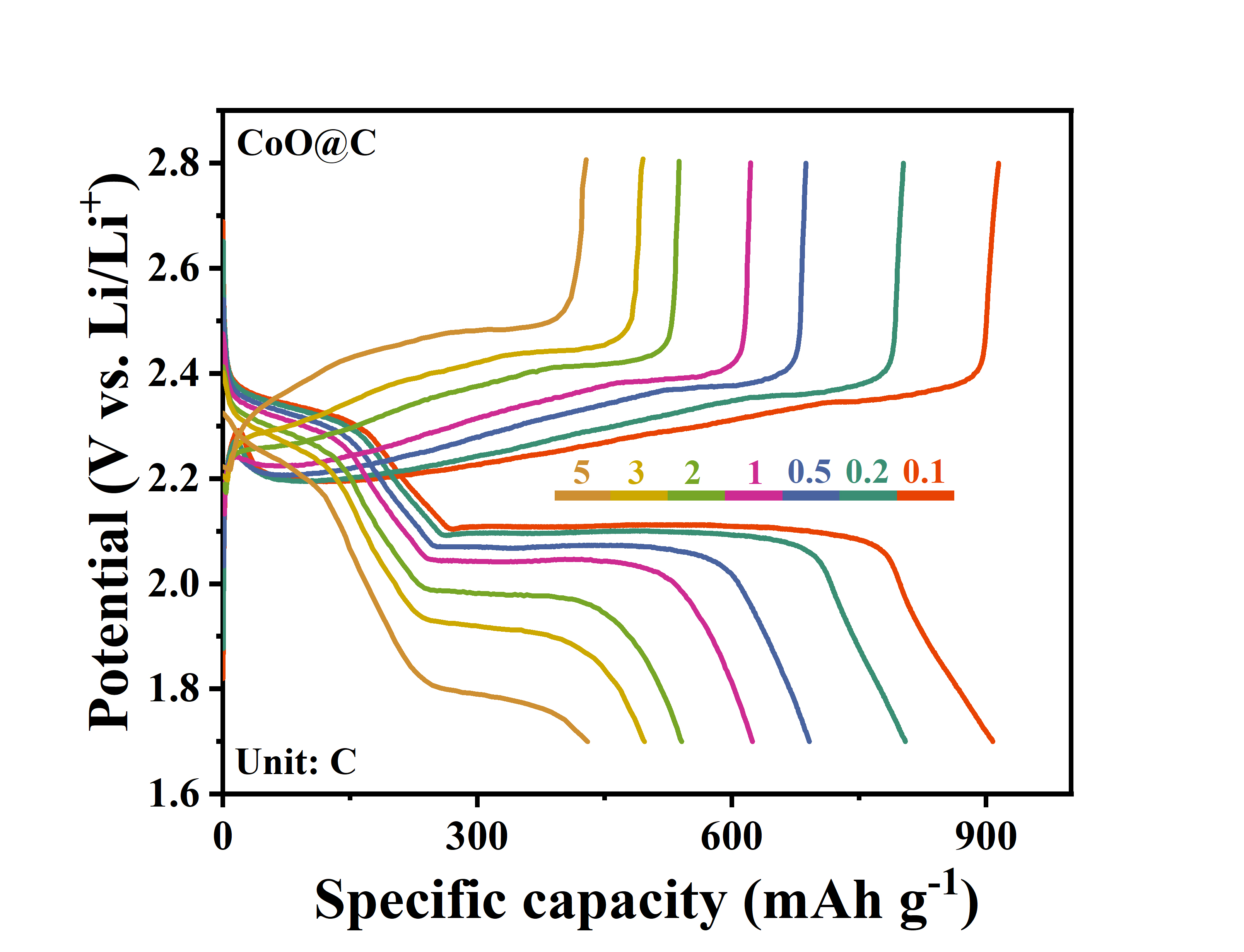


**Figure S18** Charge-discharge profiles at different rates of CoO@C.

**
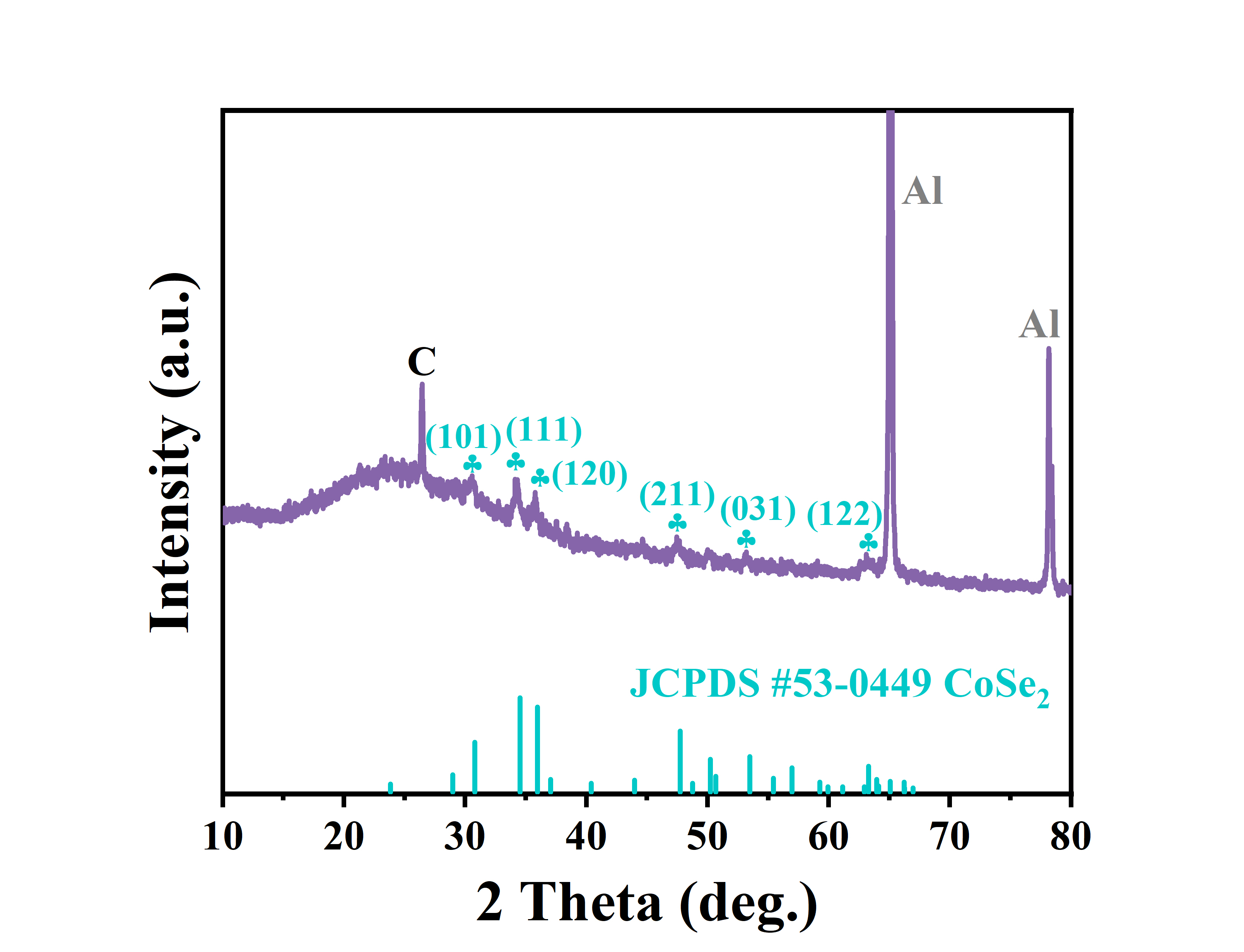
**

**Figure S19** XRD pattern of cathode sheet after cycling.

**
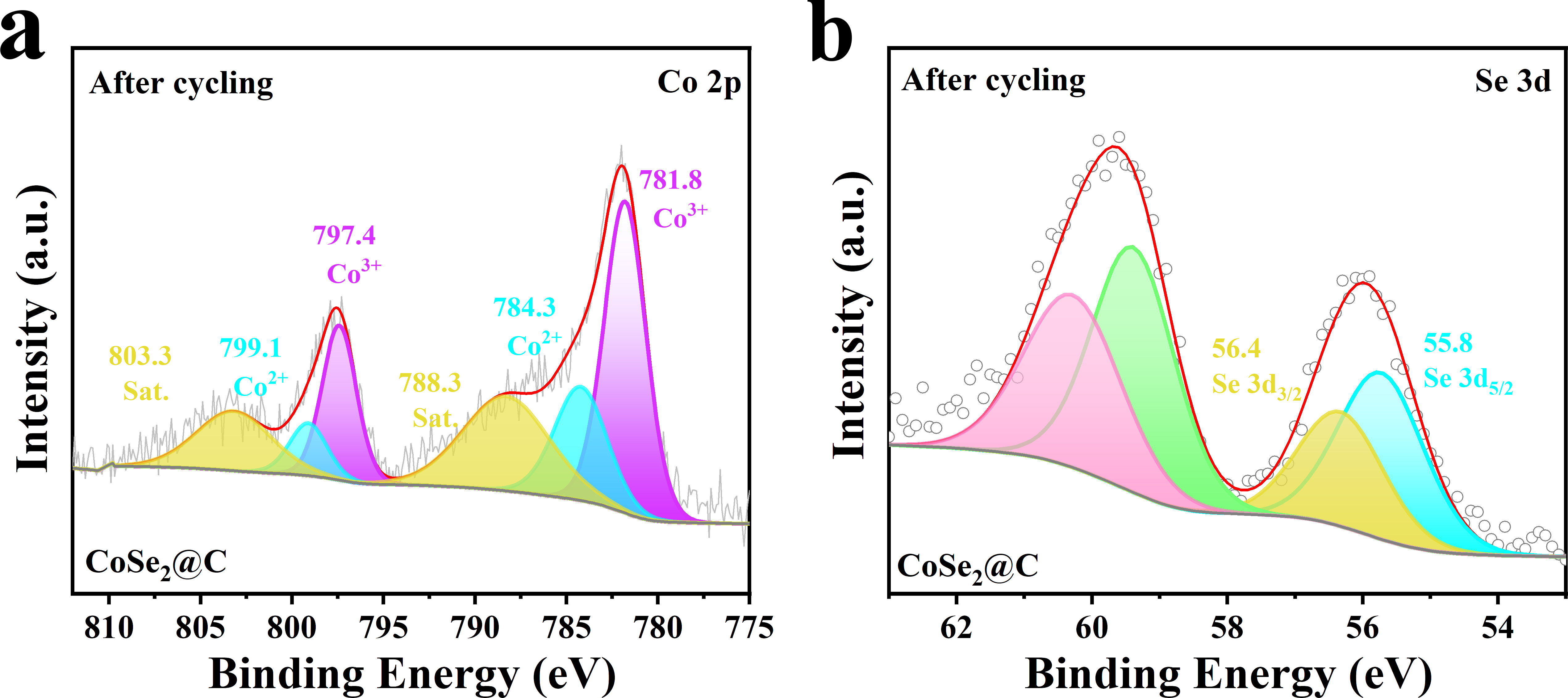
**

**Figure S20** XPS of CoSe_2_@C on the cathode sheet after cycling.

**
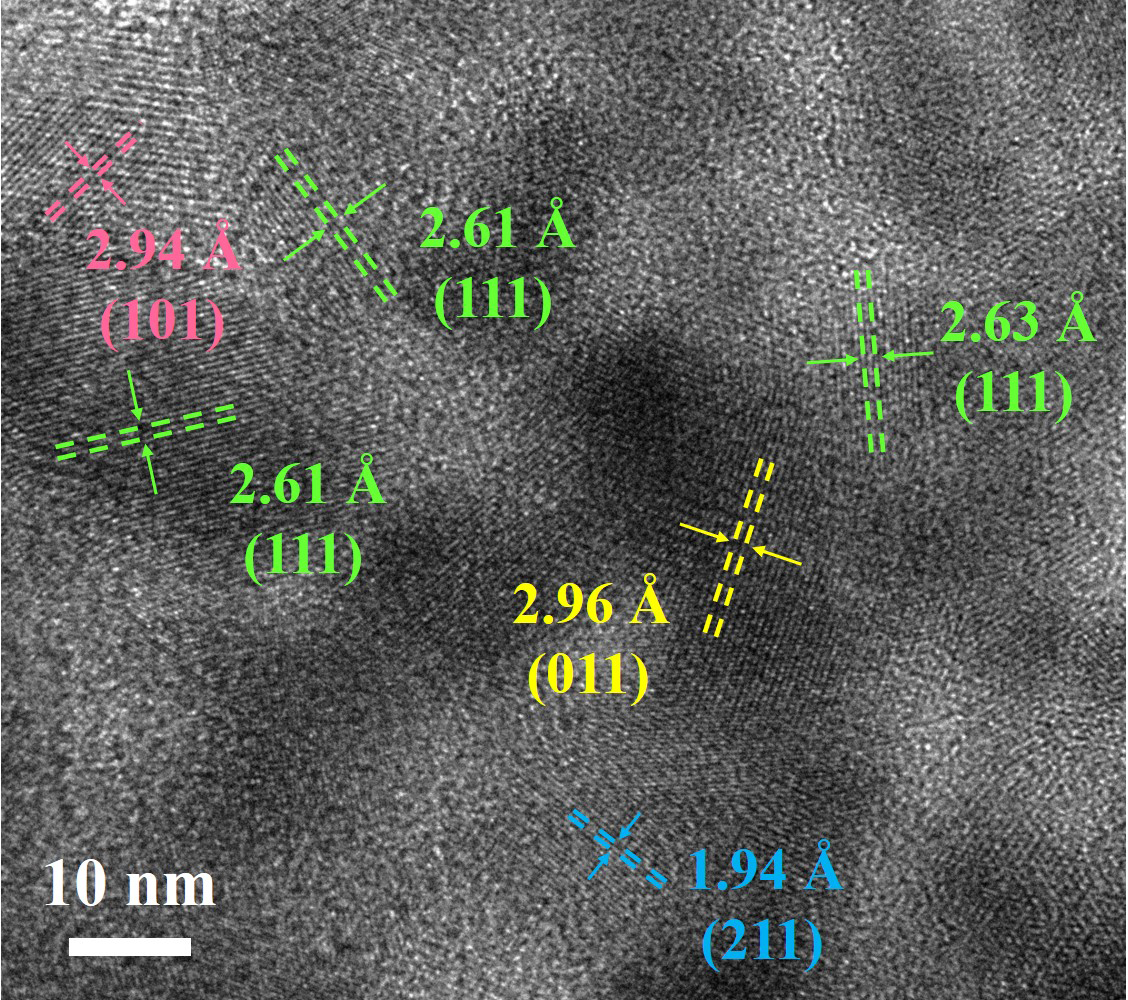
**

**Figure S21** HRTEM of CoSe_2_@C on the cathode sheet after cycling.

**
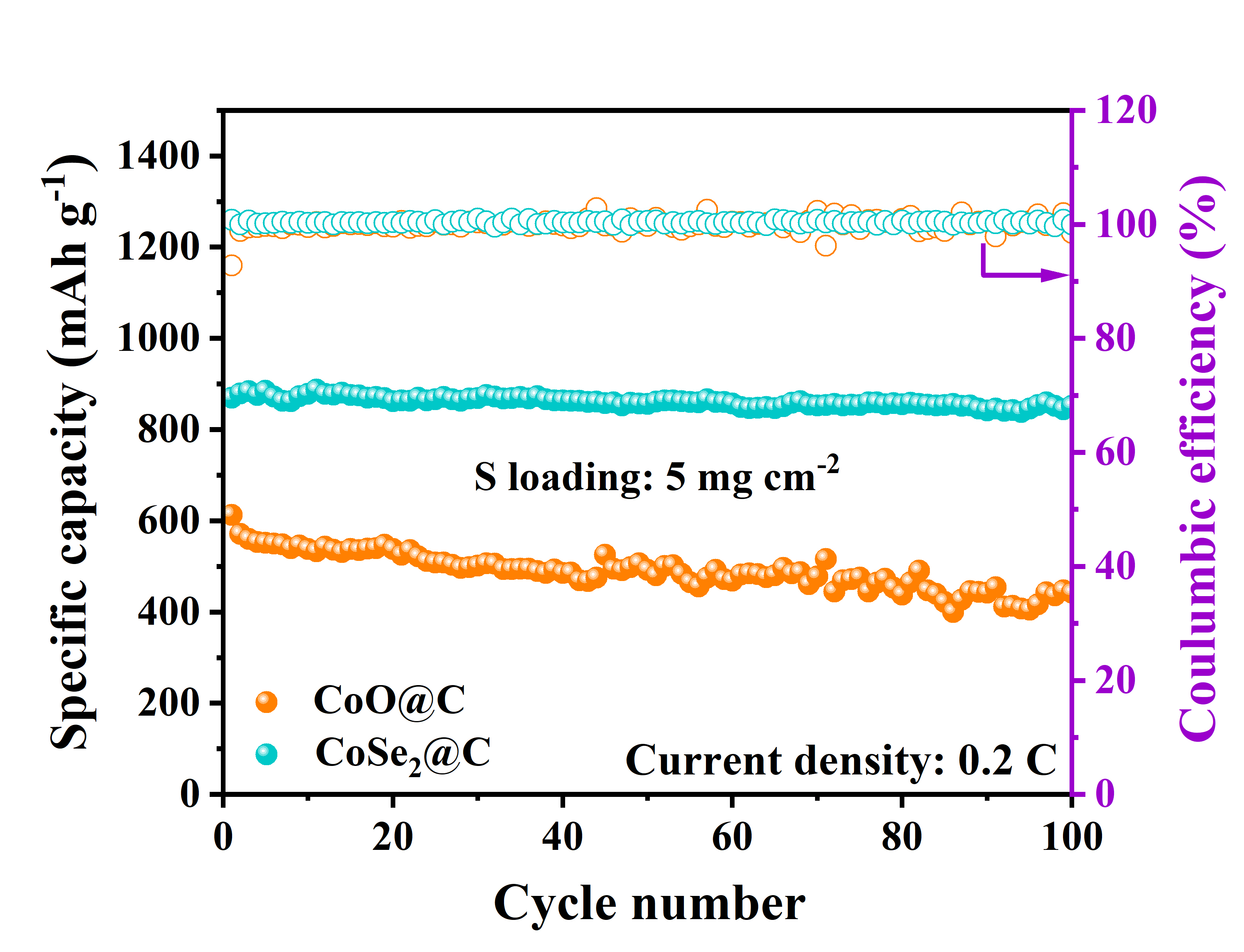
**

**Figure S22** Cycle performance at 0.2 C with the high sulfur loading of 5 mg cm^-2^.

**Table S1** The impedance value in the equivalent circuit before cycling

| **Cathode** | ***R_s_* (Ω)** | ***R_ct_*** (Ω) |
| --- | --- | --- |
| CoSe_2_@C | 1.879 | 70.30 |
| CoO@C | 2.528 | 98.74 |

**Table S2** The impedance value in the equivalent circuit after cycling

| **Cathode** | ***R_s_* (Ω)** | ***R_surf_*** (Ω) | ***R_ct_*** (Ω) |
| --- | --- | --- | --- |
| CoSe_2_@C | 1.995 | 4.250 | 6.897 |
| CoO@C | 2.409 | 5.546 | 30.700 |

**Table S3** Performance comparison of various sulfur cathode materials

| Cathode | C-rate | Capacity (mAh g^-1^) | Retention@cycle  (mAh g^-1^) | Rate/Capacity  (mAh g^-1^) | Ref. |
| --- | --- | --- | --- | --- | --- |
| CoSe_2_@C | 2 C | 931 | 666^@1000th^ | 5 C/666 | This work |
| Ni_0.1_Zn_0.1_Co_0.8_Se_2_ | 0.5 C | 843.03 | 681.64^@100th^ | 2 C/681.74 | [S1] |
| PCC@CoSe_2_ | 0.5 C | 998.3 | 602.2^@100th^ | 5 C/364 | [S2] |
| Ti_3_C_2_T*_x_*@CoSe_2_ | 0.5 C | 1032.7 | 560^@800th^ | 3 C/694 | [S3] |
| RGO-CoSe_2_ | 1 C | 1036.6 | 741.2^@400th^ | 2 C/697 | [S4] |
| CoSe_2_@CNF/CNT | 1 C | 1098.8 | 766.4^@500th^ | 3 C/915.1 | [S5] |
| CCGM | 0.5 C | 983.28 | 482.46^@300th^ | 5 C/449.45 | [S6] |
| CoSe_2_@CNF | 1 C | 1182.8 | 587.3^@400th^ | 2 C/730 | [S7] |
| o-CoSe_2_ | 1 C | 969.9 | 622.2^@300th^ | 3 C/738.4 | [S8] |
| Fe-CoSe_2_ | 1 C | 1473 | 444^@1000th^ | 2 C/827 | [S9] |
| CoSe_2_/MMC | 1 C | 940.5 | 634.8^@1000th^ | 5 C/728.2 | [S10] |
| CoSe_2_@B-C_3_N_5_-1 | 2 C | 662 | 364^@1000th^ | 4 C/554 | [S11] |
| S-CoSe_2_ | 1 C | 793.21 | 455.5^@600th^ | 4 C/582.12 | [S12] |
| CoSe_2_@NCNFs | 1 C | 828.4 | 291.4^@1000th^ | 3 C/605 | [S13] |
| THC-CoSe_2_ | 1 C | 664.4 | 540.6^@200th^ | 2 C/480 | [S14] |
| CoSe_2_/C | 1 C | 810.5 | 503.4^@400th^ | 2 C/760.5 | [S15] |

**Reference**

[S1] L. Chen, Y. Xu, G. Cao, H. M. K. Sari, R. Duan, J. Wang, C. Xie, W. Li, X. Li, *Adv. Funct. Mater.* **2022**, *32*, 2107838.

[S2] C. Wang, R. Liu, W. Liu, W. Zhu, X. Yang, Q. Wu, K. Xie, L. Shen, J. Wu, Y. Liu, L. He, Z. Chen, J. Chen, C. Zhao, X. Lin, L. Shi, J. Zhao, X. Feng, G. Wu, Y. Ma, *Adv. Funct. Mater.* **2024**, *34*, 2316221.

[S3] L. Cai, H. Ying, P. Huang, Z. Zhang, H. Tan, Q. Han, W.-Q. Han, *Chem. Eng. J.* **2023**, *474*, 145862.

[S4] L. Chen, W. Yang, J. Liu, Y. Zhou, *Nano Res.* **2019**, *12*, 2743.

[S5] J. Ao, Y. Xie, Y. Lai, M. Yang, J. Xu, F. Wu, S. Cheng, X. Wang, *Sci. China Mater.* **2023**, *66*, 3075.

[S6] F. Han, D. Yan, X. Guan, Q. Lu, S. Yin, Y. Yan, H. Zhou, P. Yang, Q. Zhang, S. Zhang, J. Xia, Y. Xing, *Energy Storage Mater.* **2024**, *71*, 103652.

[S7] Y. Zhang, Y. Li, C. Xu, D. Li, J. Cao, P. Huo, *J. Energy Storage* **2024**, *101*, 113889.

[S8] G. Liu, Q. Zeng, S. Tian, K. Tao, E. Xie, Z. Zhang, *Chem. Eng. J.* **2023**, *463*, 142416.

[S9] J. Ren, L. Zeng, J. Pan, W. Yang, C. Ding, J. Li, Q. Liu, *Adv. Sustain. Syst.* **2025**, 9, 2500056.

[S10] Y. Lv, J. Zhang, S. Liu, Y. Liu, X. Jia, J. Yang, L. Ma, Y. Wang, H. Wang, J. Li, Z. Jin, *J. Energy Storage* **2025**, 126, 117078.

[S11] Y. Zhang, M. Wang, B. Chen, W. Zeng, Y. Liu, H. Yang, J. Huang, M. Zhou, *Chem. Eng. J.* **2025**, 510, 161776.

[S12] L. Chen, K. Xue, X. Wang, R. Duan, G. Cao, S. Li, G. Zu, Y. Li, J. Wang, X. Li, *ACS Appl. Mater. Interfaces* **2024**, 16, 48639.

[S13] H. He, W. Lei, L. Liu, X. Zhang, J. Zhang, Z. Shu, Z. Hu, L. Yang, Y. Li, X. Hu, *Chem. Eng. J.* **2024**, 489, 151501.

[S14] B. Guo, Q. Ma, L. Zhang, T. Yang, D. Liu, X. Zhang, Y. Qi, S.-J. Bao, M. Xu, *Chem. Eng. J.* **2021**, 413, 127521.

[S15] L. He, D. Yang, H. Zhao, L. Wei, D. Wang, Y. Wang, G. Chen, Y. Wei, *Chem. Eng. J.* **2022**, 440, 135820.
